# Magneto-Induced Hyperthermia and Temperature Detection in Single Iron Oxide Core-Silica/Tb^3+^/Eu^3+^(Acac) Shell Nano-Objects

**DOI:** 10.3390/nano12183109

**Published:** 2022-09-07

**Authors:** Karina Nigoghossian, Basile Bouvet, Gautier Félix, Saad Sene, Luca Costa, Pierre-Emmanuel Milhet, Albano N. Carneiro Neto, Luis D. Carlos, Erwan Oliviero, Yannick Guari, Joulia Larionova

**Affiliations:** 1 ICGM, University of Montpellier, CNRS, ENSCM, 34000 Montpellier, France; 2 Centre de Biologie Structurale (CBS), University of Montpellier, CNRS, INSERM, 34000 Montpellier, France; 3 Phantom-G, Physics Department and CICECO—Aveiro Institute of Materials, University of Aveiro, 3810-193 Aveiro, Portugal

**Keywords:** luminescence, lanthanide coordination complexes, nanothermometry, magnetic iron oxide nanoparticles, magnetothermia, hybrid nano-systems

## Abstract

Multifunctional nano-objects containing a magnetic heater and a temperature emissive sensor in the same nanoparticle have recently emerged as promising tools towards personalized nanomedicine permitting hyperthermia-assisted treatment under local temperature control. However, a fine control of nano-systems’ morphology permitting the synthesis of a single magnetic core with controlled position of the sensor presents a main challenge. We report here the design of new iron oxide core–silica shell nano-objects containing luminescent Tb^3+^/Eu^3+^-(acetylacetonate) moieties covalently anchored to the silica surface, which act as a promising heater/thermometer system. They present a single magnetic core and a controlled thickness of the silica shell, permitting a uniform spatial distribution of the emissive nanothermometer relative to the heat source. These nanoparticles exhibit the Tb^3+^ and Eu^3+^ characteristic emissions and suitable magnetic properties that make them efficient as a nanoheater with a Ln^3+^-based emissive self-referencing temperature sensor covalently coupled to it. Heating capacity under an alternating current magnetic field was demonstrated by thermal imaging. This system offers a new strategy permitting a rapid heating of a solution under an applied magnetic field and a local self-referencing temperature sensing with excellent thermal sensitivity (1.64%·K^−1^ (at 40 °C)) in the range 25–70 °C, good photostability, and reproducibility after several heating cycles.

## 1. Introduction

Nanoparticle induced hyperthermia is one of the promising treatments in the development of personalized nanomedicine that aims to improve the effectiveness and safety of conventional treatments and diagnosis [1,2]. Although the use of alternating magnetic fields that induce magnetic iron oxide nanoparticles to heat has already been approved in Europe to treat glioblastoma (NanoTherm^®^, Magforce, Berlin, Germany) [3], several progresses are needed to control the temperature and avoid overheating conducting that damages surrounding healthy tissues. In this connection, there is a lack of appropriate tools for fine and local temperature sensing because the insertion of needle thermocouples near the target area is an invasive approach with spatial resolution limited to the size of the probe [4]. A recently emerged all-in-one approach represents a significant breakthrough in this area [5,6,7]. It consists in the design of smart multifunctional nanoplatforms combining a magnetic heater able to generate local heat under an applied alternating current (AC) magnetic field with an emissive sensor to monitor the local temperature rise. The employment of emissive sensors is highly promising because they are able to remotely provide temperature-dependent signals leading to fine thermal imaging during the hyperthermia process with high resolution. The sensor function may be achieved by organic dyes [5], Ln^3+^-based complexes [8,9,10], silver sulfide nanoparticles [11,12], or ceramic Ln^3+^ containing nano-objects [13,14,15]. Magnetic iron oxide nanoparticles of an appropriate size have proved their efficiency as hyperthermia heater [5,15,16,17,18,19,20,21,22,23,24,25].

The ultimate goal therefore consists of the fine measurements of a local temperature elevation in the single magnetic heater/emissive sensor nano-system. However, the design of such nano-objects is very challenging, as it requires a perfect mastery of nanoparticles’ morphology and implies the synthesis of structurally well-defined monodisperse hybrid nano-systems. In this connection, several reported works may be classified in two strategies consisting of: (i) an encapsulation of heater (IONPs) and sensor nanoparticles in large mesoporous silica nano-objects [26] or phospholipidic [12] or polymeric capsules [13], or (ii) synthesis of core-shell systems, where the iron oxide core has been surrounded by a shell of polymers [5,9,14] or silica [8] containing luminescent species. However, to the best of our knowledge, the reported works did not achieve multifunctional magneto-luminescent nanoparticles with a good control of their morphology. Indeed, each noted example presents its own advantages and drawbacks, but in the most of them, except for three works [5,26,27], several aggregated magnetic nanoparticles are enclosed into the not-well-controlled shell, which is far from optimal for the efficient single heater/thermometer nano-system. Moreover, sensor species are often physically dispersed in the shell, which presents a risk of their release. Only one article reported on the covalent attachment of organic dye on the surface of single magnetic nanoparticles, which is not a reversible nor a self-referenced method for application in hyperthermia [5].

**Scheme 1:**
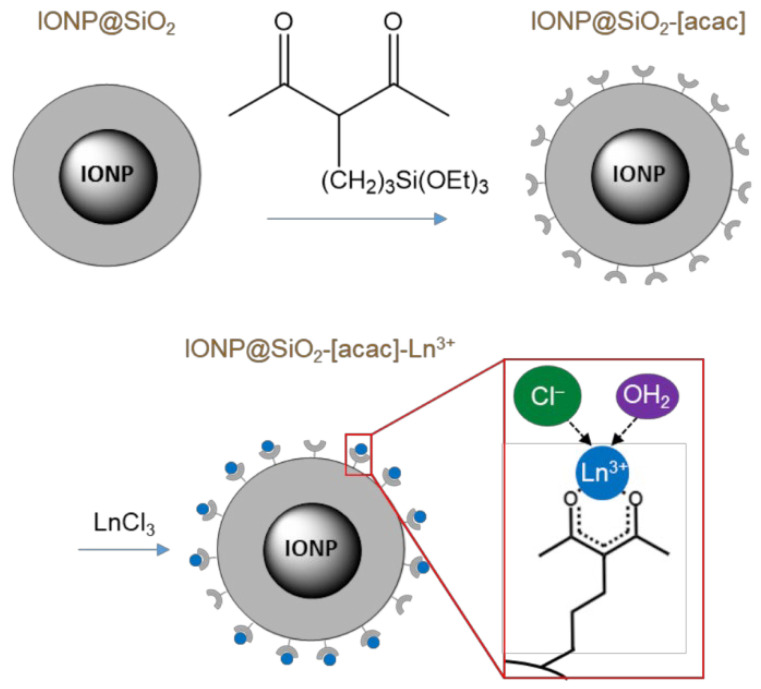
Synthesis of multifunctional heater–temperature sensor nanoplatform as IONP core@SiO_2_-*acac*/Ln^3+^ (Ln^3+^ = Tb^3+^/Eu^3+^) shell nano-objects. The Ln^3+^ complexes were covalently grafted at the surface of a silica shell functionalized with *acac* groups.

In this work, we report new multifunctional core@shell nano-objects containing a single magnetic IONP core surrounded by a silica shell with covalently attached Tb^3+^/Eu^3+^ based complexes acting as a self-referencing emissive sensor (Scheme 1). The silica shell has been chosen due to its protective role, biocompatibility, the possibility of the shell’s thickness control, and the well-developed surface chemistry allowing covalent attachment of targeted functions.

To the best of our knowledge, only two works noted the use of silica coating for the design of magnetic heater@silica shell platform with emissive temperature sensor [8,27]. In the article reported by A. Pralle’s group, an organic dye was loaded into the silica shell during the coating of MnFe_2_O_4_ nanoparticles [27]. In the second work of L. D. Carlos and colleagues, luminescent Ln^3+^ complexes were included in the silica shell to act as a self-referencing thermometer [8]. However, this latter system presented two of the aforementioned drawbacks linked with difficulty of the morphology control and a physical dispersion of the luminescent species. We employed here Tb^3+^/Eu^3+^-acetylacetonate (*acac*) moiety as emissive sensors due to numerous expected advantages: (i) they may be considered as self-referencing emissive sensors [28,29,30,31,32], (ii) they are expected to present high photothermal stability [8,9], (iii) they are expected to show good relative thermal sensitivity with the possibility to tune the working temperature range, and (iv) they can be covalently anchored to the silica shell through a modified *acac* ligand. The magnetic heater/emissive sensor nano-system obtained in this work presents a perfectly controlled core@shell morphology with a well definite IONP core and a silica shell with adjustable thickness comprising covalently grafted Tb^3+^/Eu^3+^-*acac* complexes. It is able to induce an important temperature rise under an applied AC magnetic field and to measure the temperature by emissive self-referencing sensing.

## 2. Materials and Methods

### 2.1. Materials

Ferric hydroxide oxide (hydrated, 30–50 mesh), oleic acid (90%), oleylamine (90%), n-docosane (99%), ammonia (30%), tetraethylorthosilicate (TEOS, 99%), (3-chloropropyl)-triethoxysilane (95%), (3-iodopropyl)trimethoxysilane (95%), sodium iodide, potassium tertbutoxide (tBuOK), tert-butanol (tBuOH), europium(III) chloride hexahydrate (99.9%, trace metals basis), terbium(III) chloride hexahydrate (99.9%, trace metals basis), acetylacetone, pentane, diethyl ether, cyclohexane, acetone, and ethanol were purchased from Merck. Triton X-100 and 1-hexanol (99%) were purchased from Alfa Aesar (Haverhill, MA, USA).

### 2.2. Synthesis

#### 2.2.1. Synthesis of Iron Oxide Nanoparticles

The synthesis of the iron oxide nanoparticles (IONP/OA/OA) of ca. 26 nm was performed following a thermal decomposition method reported elsewhere with minor modifications [33]. Typically, the reaction occurs at high temperature by mixing an iron precursor (FeO(OH)) with a coordinating ligand (oleic acid) and a non-coordinating reaction solvent with a high-boiling point (*n*-docosane). First, a flask containing a mixture of FeO(OH) (2 mmol, 0.18 g), oleic acid (10 mmol, 3.2 g), and *n*-docosane (5.0 g) was connected to a Schlenk line to remove moisture and oxygen for 30 min at room temperature under vacuum and magnetic stirring. Subsequently, the flask was heated to 350 °C under argon flow at a heating rate of 10 °C/min. The solution was maintained at 350 °C for a further 90 min under stirring and argon flow. After this period, the heating source was removed. When the temperature of the solution reached 50 °C, pentane (15 mL) was added to the obtained nanoparticles. The nanoparticles were washed three times by dispersing in diethyl ether, followed by precipitation with ethanol (1:1 *v*/*v*), and then recovered using centrifugation (20,000 rpm, 10 min). Oleylamine (200 µL) was added to the collected material. The resultant oleate/oleylamine-capped IONPs (IONP/OA/OA) were finally dispersed in cyclohexane (15 mL).

#### 2.2.2. Silica Coating of Iron Oxide Nanoparticles

**Figure 1:**
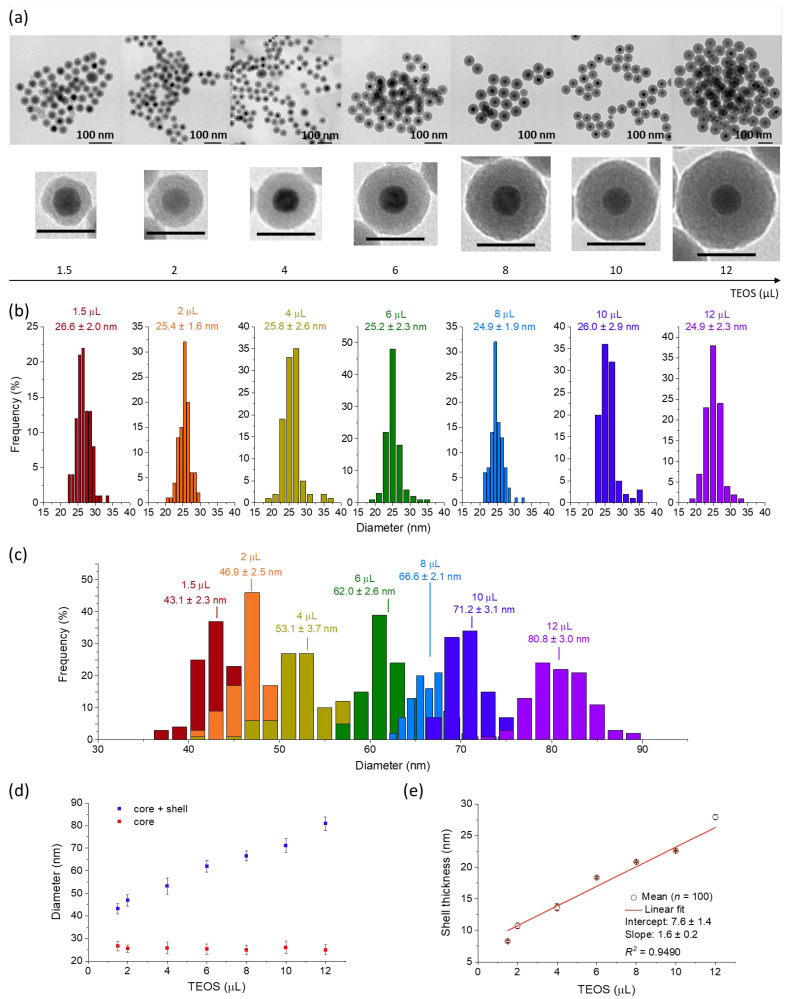
(**a**) TEM images of IONP@SiO_2_ prepared by adding different amounts of TEOS along with their magnified images of single nanoparticles (scale bars represent 50 nm). Size distributions (*n* = 100) of (**b**) IONP core and (**c**) whole core-shell IONP@SiO_2_. (**d**) Core and core@shell diameters as a function of the added TEOS amount and (**e**) shell thickness as a function of the added TEOS amount. For (**d**,**e**), the error bars correspond to standard error of mean, *n* = 100.

The obtained hydrophobic IONP/OA/OA was coated with a silica shell. The adapted method consists of encapsulating the nanoparticles in a reverse microemulsion [34]. First, Triton X-100 (1.77 g), 1-hexanol (1.60 mL), and cyclohexane (7 mL) were vigorously mixed. After 1 h of magnetic stirring, the IONPs solution (0.5 mg in 0.5 mL of cyclohexane) was rapidly added. Stirring was continued for a further 1 h. Ammonia (6%, 0.5 mL) was added to form a water-in-oil microemulsion. The emulsion was stirred for 1 h to ensure a uniform distribution of nanocrystals within the micelles. Different amounts of TEOS (from 1.5 to 12 µL) were added in order to modulate the silica shell thickness from ca. 8 to 28 nm (see Figure 1a and Table S1), and the mixture was stirred at 500 rpm for 24 h. The nanoparticles were collected by centrifugation (20,000 rpm, 15 min), then washed with ethanol and recovered by centrifugation. The IONP@SiO_2_ core-shell nanoparticles were stored in 2 mL of ethanol until further use. The nanoparticles with silica shell of ca. 11 nm (2 µL of added TEOS) were chosen for further experiments because they combined the thinnest silica shell and a good organization of shell around the IONP core (Table S1). Therefore, their synthesis was successfully scaled up (×100 times) for further grafting of complexes.

#### 2.2.3. Synthesis of Acetylacetonate-Functionalized Organosilane

The synthesis of compound [CH_3_C(O)]_2_CH(CH_2_)_3_-Si(OCH_2_CH_3_)_3_ (*acac*-silane) was performed from I(CH_2_)_3_Si(OCH_2_CH_3_)_3_ according with the method previously described [35]. First, I(CH_2_)_3_Si(OCH_2_CH_3_)_3_ was synthesized by reacting (3-chloropropyl)-triethoxysilane (30.0 g, 124.7 mmol) and sodium iodide (28.1 g, 187.1 mmol) at reflux in acetone (200 mL) for 72 h. The solvent was removed under vacuum and pentane was then added. The resulting suspension was filtered to remove the salts. The solvent was removed under vacuum. The product was distilled under reduced pressure and rendered a colorless oil. Second, [CH_3_C(O)]_2_CH(CH_2_)_3_-Si(OCH_2_CH_3_)_3_ was prepared by adding acetylacetone (15.0 g, 150 mmol) to a solution of *t*BuOK (11.2 g, 100 mmol) in *t*BuOH (100 mL) at room temperature. After stirring for 10 min, (3-iodopropyl)trimethoxysilane (33.2 g, 100 mmol) was added. The mixture was stirred and heated under reflux for 20 h. The solvent was removed under vacuum and the residue dissolved in pentane (200 mL). After filtration under argon, the filtrate was concentrated. The residual clear yellow liquid was distilled, yielding a colorless liquid.

#### 2.2.4. Acetylacetonate-Functionalization of Silica-Coated Iron Oxide Nanoparticles

The grafting of acetylacetonate (*acac*) groups on the silica surface of nanoparticles was performed by dispersing the IONP@SiO_2_ (*ca*. 11 nm-silica shell) in toluene (33 mg in 40 mL) at 110 °C under reflux, and adding the *acac*-silane (100 µL). After overnight reaction, the *acac*-functionalized nanoparticles (IONP@SiO_2_-*acac*) were washed three times with ethanol (20,000 rpm, 20 min) and finally redispersed in ethanol (20 mL).

#### 2.2.5. Complexation of Ln^3+^ with Acac-Functionalized Iron Oxide Nanoparticles

The complexation of lanthanide ions occurred by adding their chlorides solutions in ethanol (20 mM) in a defined molar ratio Tb^3+^:Eu^3+^ = 95:5 (0.95 mmol of TbCl_3_·6H_2_O and 0.05 mmol of EuCl_3_·6H_2_O) to the IONP@SiO_2_-*acac* in ethanol (4.2 mg in 2.5 mL). The mixture was stirred at 80 °C under reflux overnight. The Ln^3+^-containing nanoparticles (IONP@SiO_2_-*acac*/Ln^3+^) were washed three times with ethanol (20,000 rpm, 20 min) and finally redispersed in ethanol (2 mL). For analyses on powdered samples, the nanoparticle samples were collected using centrifugation (20,000 rpm, 10 min), then dried under normal air conditions to constant weight, followed by vacuum at room temperature.

Atomic percentages in IONP@SiO_2_-*acac*/Tb^3+^/Eu^3+^ detected by SEM-EDX: % Tb 3.87, % Eu 0.22, % Si 66.68, % Fe 28.54, % Cl 0.68. IR (InfraRed) band assignments are given in Table S1.

### 2.3. Characterizations

The size and shape of the obtained nanoparticles were observed by transmission electronic microscopy (TEM) at 100 kV (JEOL 1200 EXII). The images were analyzed using the ImageJ software to measure the size of nanoparticles (*n* = 100), assessed as the Feret’s diameter. The Origin software (Version 2020, OriginLab Corporation, Northampton, MA, USA) was used for statistical analysis on nanoparticle sizes. The size distributions of the nanoparticles were obtained using the frequency counts analysis of Origin software and given as an average diameter ± standard deviation. The average diameter corresponded to the mean values of measured Feret’s diameter. The standard deviation (s) has been classically calculated by the Origin software as:

(1) $$s=\sqrt{\sum_{i=1}^{n}\omega_{i}{\left(x_{i}-\bar{x}\right)}^{2}/d},\;$$

where n is the total number of data points (*n* = 100),$\;x_{i}$ is the ith sample, $\omega_{i}\;$is the ith weight, and d=n−1.

High resolution transmission electron microscopy (HRTEM) images were acquired using a JEOL 2200 FS (FEG) operated at 200 kV with a Gatan UltraScan 4000 (4 k × 4 k) CCD camera. The elemental mapping was recorded in scanning mode (STEM) with an Oxford Instruments XMaxN 100 TLE (100 mm^2^, windows less) EDX detector. Dynamic light scattering (DLS) measurements (Zetasizer Nano-series Malvern instrument, model ZEN3600) were used to determine the particle hydrodynamic diameter of colloidal suspensions.

The crystal structural characteristics were investigated by X-Ray diffraction (XRD) using a PANalytical X’Pert Powder analytical diffractometer mounted in a Debye−Scherrer configuration and equipped with Cu radiation (λ = 1.5418 Å) on powdered samples. Infrared (IR) spectra using attenuated total reflectance (ATR-IR) were recorded using powdered samples with a PerkinElmer Spectrum Two FT-IR Spectrometer. Quantifications of Tb, Eu, Si, and Fe elements were performed by using a scanning electron microscope and energy dispersive X-ray analysis (SEM-EDX) on an FEI Quanta FEG 200 instrument. The powders were deposited on an adhesive carbon film and analyzed under high vacuum. The quantification of the heavy elements was carried out with the INCA software, with a dwell time of 3 µs. Thermogravimetric analyses (TGA) under air atmosphere were obtained on powders with a thermal analyzer STA 409 Luxx^®^ (Netzsch) in the temperature range 20–800 °C at a heating speed of 5 °C min^−1^. Brunauer–Emmett–Teller (BET) surface area was obtained with N_2_ sorption isotherms performed at 77 K using a Micromeritics Tristar unit (USA). Prior to the analysis, samples were degassed for 12 h at 100 °C under primary vacuum.

The emission and excitation spectra were recorded at 295 K using a spectrofluorimeter Edinburgh FLS-920. The excitation source was a 450 W Xe arc lamp. The spectra were corrected for detection and optical spectral response of the spectrofluorimeter. To obtain the thermal calibration curves, the emission spectra were measured at different temperatures in a Peltier-based temperature-controlled cuvette holder coupled to the spectrofluorometer. The colloidal solutions were maintained under magnetic stirring during thermal calibration measurements. A period of 100 s was given to allow the temperature to stabilize. Temperature accuracy was ±0.06 °C.

Magnetic measurements were performed using a SQUID MPMS-XL magnetometer working in the temperature range 1.8–350 K up to 7 T by using static (direct current (DC)) and dynamic (alternating current (AC)) modes in powdered samples. The data were corrected for the sample holder. The AC magnetic susceptibility measurements were carried out in the presence of a 3.5 Oe oscillating field in zero or applied external DC field. Temperatures associated to relaxation times (1/(2πf)) were extracted from the out-of-phase AC magnetic susceptibility, and the fit of the relaxation times was realized by using the Arrhenius equation:

(2) $$\tau =\tau_{0}\exp\left(\frac{E_{a}}{k_{B}T}\right)\to \ln\left(\tau \right)=\ln\left(\tau_{0}\right)+\frac{E_{a}}{k_{B}T},$$

where *E_a_* is the barrier energy in cm^−1^; and the Vogel–Fulcher equation:

(3) $$\tau =\tau_{0}\exp\left(\frac{E_{a}}{k_{B}\left(T-T_{0}\right)}\right)\to \ln\left(\tau \right)=\ln\left(\tau_{0}\right)+\frac{E_{a}}{k_{B}\left(T-T_{0}\right)}$$

where *T_0_* is an additional parameter which takes into account dipolar interactions [36].

Magnetothermia experiences were realized using an AC magnetic field generator (UltraFlex) at 350 kHz. The generating magnetic field is around 20 mT. The samples were in liquid state and isolated with polystyrene. The temperature of the liquid surface was measured using an OPTRIS PI 450 thermal camera.

### 2.4. Theoretical Procedures

The intramolecular energy transfer rates (IET) from the first triplet state (T_1_, ca. 27,000 cm^–1^ [37]) to the Ln^3+^ ion can be calculated considering the dipole–dipole ($W_{d-d}$), dipole–multipole ($W_{d-m}$), and exchange ($W_{ex}$) mechanisms [38,39,40]:

(4) $$W_{d-d}=\frac{S_{L}{\left(1-\sigma_{1}\right)}^{2}}{\left(2J+1\right)G}\frac{4\pi }{\hbar }\frac{e^{2}}{R_{L}^{6}}\sum_{\lambda }\Omega_{K}^{FED}\langle \psi^{\prime }J^{\prime }∥U^{\left(K\right)}∥\psi J\rangle^{2}F$$

(5) $$W_{d-m}=\frac{S_{L}}{\left(2J+1\right)G}\frac{2\pi e^{2}}{\hbar }\sum_{K}\left(K+1\right)\frac{\langle r^{K}{}^{2}\rangle }{{\left(R_{L}^{K+2}\right)}^{2}}\langle f∥C^{\left(K\right)}∥f\rangle^{2}{\left(1-\sigma_{K}\right)}^{2}\langle \psi^{\prime }J^{\prime }∥U^{\left(K\right)}∥\psi J\rangle^{2}F$$

(6) $$W_{ex}=\frac{{\left(1-\sigma_{0}\right)}^{2}}{\left(2J+1\right)G}\frac{8\pi }{\hbar }\frac{e^{2}}{R_{L}^{4}}\langle \psi^{\prime }J^{\prime }∥S∥\psi J\rangle^{2}\sum_{m}{\left|\langle \phi |\sum_{j}\mu_{z}\left(j\right)s_{m}\left(j\right)|\phi^{*}\rangle \right|}^{2}F$$

where $R_{L}$ is the donor–acceptor states distance (assumed a reasonable value of 4.0 Å for both Eu^3+^ and Tb^3+^ complexes), and $\Omega_{K}^{FED}$ are the intensity parameters with the contribution of the forced electric dipole mechanism (Judd-Ofelt theory). The quantities $\langle \psi^{\prime }J^{\prime }∥U^{\left(K\right)}∥\psi J\rangle$ are reduced matrix elements, and their values are tabulated in [41]. The $S_{L}$ term is the dipole strength of the donor state T_1_ involved in IET. The $\langle r^{K}\rangle$ are the 4*f* radial integrals [42,43], G is the state degeneracy (equal 3 for T_1_), and $\left(1-\sigma_{K}\right)$ are the shielding factors [44,45,46]; $s_{m}$ is the spin operator in the ligand, $\mu_{z}$ is the dipole operator (its z-component), and $\langle \psi^{\prime }J^{\prime }∥S∥\psi J\rangle$ is the reduced matrix elements of the spin operator for the Ln^3+^ side, which were calculated previously using free-ion wavefunctions in the intermediate coupling scheme [40].

The F (Equations (3)–(5)) is the spectral overlap factor that contains the energy mismatch conditions. Once the bandwidth at half-height for the ligands ($\gamma_{L}\approx 3000$ cm^−1^) is much larger than the lanthanides ($\gamma_{Ln}\approx 300$ cm^−1^), $\gamma_{L}\gg \gamma_{Ln}$, this factor can simply be obtained as follows [38]:

(7) $$F=\frac{1}{\hbar \gamma_{L}}\sqrt{\frac{\ln\left(2\right)}{\pi }}e^{-{\left(\frac{\delta }{\hbar \gamma_{L}}\right)}^{2}\ln\left(2\right)}$$

where the δ is the energy difference between the T_1_ donor state (D) and the lanthanide ion acceptor state, $\delta =E_{D}-E_{Ln}$.

The forward energy transfer rates ($w^{T}$, T_1_ → Ln^3+^) involving the Ln^3+^ as acceptor are calculated by the sum over all mechanisms in the same pathway labelled as n:

(8) $$w^{T}\left(n\right)=W_{d-d}\left(n\right)+W_{d-m}\left(n\right)+W_{ex}\left(n\right)$$

The backward energy transfer rates ($w_{b}^{T}$, Ln^3+^ → T_1_), that is, the energy returned from acceptor to donor state, are obtained with the same above equations, except for multiplying the energy mismatch conditions factors F (Equation (S6)) by the Boltzmann’s factor when δ<0,

(9) $$F_{b}=Fe^{-\left(\frac{\left|\delta \right|}{k_{B}T}\right)}$$

where T is the temperature and $k_{B}$ is Boltzmann’s constant.

The total IET rates ($W^{T}$ and $W_{b}^{T}$) are obtained from the sum over all individual w contributions:

(10) $$W^{T}=\sum_{n}w^{T}\left(n\right)$$

(11) $$W_{b}^{T}=\sum_{n}w_{b}^{T}\left(n\right)$$

The calculated rates involving the T1 state at room temperature are presented in Table S2 (Eu^3+^) and Table S3 (Tb^3+^).

The thermal behavior of the energy transfer rates is calculated using Boltzmann’s factor, and, in the case of Eu^3+^, the thermally coupled populations of the levels 7F0 and 7F1 are also considered. See [38,39,40,47,48] and references therein for more details on the IET rate calculations.

## 3. Results

### 3.1. Morphological and Structural Characterizations

The synthesis of IONP@SiO_2_-*acac*/Tb^3+^/Eu^3+^ nano-objects was performed using a three-step approach consisting first of the synthesis of core@shell IONP@SiO_2_ nanoparticles, the further covalent grafting of the *acac* ligand in the silica pores, and third, the coordination of the latter to the Tb^3+^/Eu^3+^ ions (Scheme 1).

The pristine magnetic IONPs of ca. 26 nm were prepared by a conventional thermal decomposition method at high temperature (350 °C) by mixing the iron precursor (FeO(OH)) with oleic acid (OA) and oleylamine (OA) as stabilizing agents and using *n*-docosane as a solvent with a high boiling point [33]. The TEM images (Figure S1a, Supporting Information (SI)) of oleate/oleylamine-capped IONPs (IONP/OA/OA) show spherical nanoparticles with a narrow size distribution presenting an average diameter of 25.5 ± 1.8 nm, as calculated from the core size distribution from TEM measurements (Figure S1, SI). A hydrodynamic diameter determined from the DLS measurements, which takes into account the presence of organic molecules anchored on the surface, is equal to 31.3 ± 9.7 nm (Figure S1c, SI).

**Figure 2:**
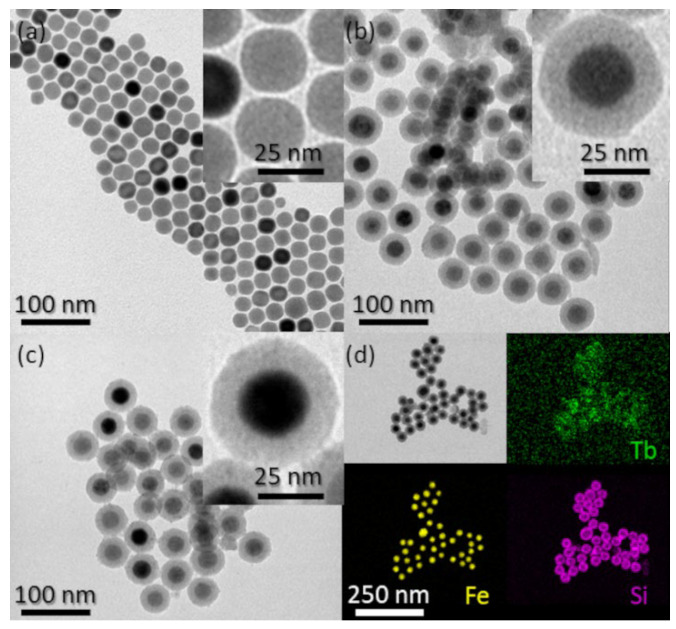
TEM images of: (**a**) IONP/OA/OA, (**b**) IONP@SiO_2_-*acac*, and (**c**) IONP@SiO_2_-*acac*/Tb^3+^/Eu^3+^. (**d**) STEM-Bright Field image (BF) and STEM-EDX elemental mapping of IONP@SiO_2_-*acac*/Tb^3+^/Eu^3+^ with topochemical distribution of Tb (green), Fe (yellow), and Si (pink).

The powder XRD pattern of the obtained sample (Figure S1d, SI) shows the main reflections attributed to the Fe_3_O_4_ phase. The peak at 2θ = 36.6° also reveals the presence of FeO phase, which suggests an incomplete oxidation of FeO at the core and an outer layer of Fe_3_O_4_ [49]. Note, however, that the presence of γ-Fe_2_O_3_ cannot be totally excluded. The subsequent silica coating of IONPs with a controlled silica shell of different thicknesses was performed through an optimized Stöber process in a reverse microemulsion system [34]. Note that the possibility to modulate the thickness of the silica shell around the iron oxide nanoparticles core has been described in the literature, but in different synthetic conditions and with different IONP cores [50]. In our system, the silica shell thickness can be linearly modulated between 8.3 ± 0.2 and 27.9 ± 0.4 nm by varying the silica precursor (TEOS) amount from 1.5 to 12 µL while keeping the IONP core size and spherical shape unmodified (Figure 1a, Table S1). The shell thicknesses (Figure 1e, Table S1) were determined from the TEM measurement-based particle size distributions as the difference between the average diameters of whole nanoparticles (Figure 2c, Table S1) and their diameters of the IONP core (Figure 2b, Table S1). All synthesized core@shell nanoparticles present a uniform and individual coating of single IONP core, except the thinner silica shell of ca. 8 nm (1.5 µL of TEOS). For the latter, some inhomogeneities of the silica shell organization around the core have been detected.

Therefore, IONP@SiO_2_ with the silica shell of ca. 11 nm (2 µL of TEOS) combining the thinner silica shell with a uniform coating of a single IONP core were selected for further grafting of Ln^3+^-based temperature sensor because of (i) the shortest distance between the Ln^3+^ based temperature sensor anchored to the silica shell and the IONP heater for the sake of an accurate temperature determination, and (ii) the expected lowest insulation effect of silica, which can reduce the magnetothermal heating [51]. First, a successful scale-up synthesis (×100) of these nanoparticles were performed. The as-obtained nanoparticles present very closed characteristics (Figure 2). Next, a covalent grafting of [CH_3_C(O)]_2_CH(CH_2_)_3_-Si(OCH_2_CH_3_)_3_ (*acac*-silane) on the silica surface of the IONP@SiO_2_ nanoparticles through the silane group and the further coordination of the *acac* moiety to Tb^3+^/Eu^3+^-ions (ratio Tb^3+^/Eu^3+^ = 19) was performed to achieve IONP@SiO_2_-*acac*/Tb^3+^/Eu^3+^ nano-objects (see Section 2.2.5 for the experimental details). The amount of *acac* ligands grafted to the silica surface of 15.2% was determined by the TGA analysis (Figure S2, Supporting Information). The atomic ratio of Fe, Si, Cl, Tb, and Eu in IONP@SiO_2_-*acac*/Tb^3+^/Eu^3+^ were determined by SEM-EDX analysis (see Section 2.2.5), which allowed us to calculate the ratio Ln^3+^:*acac* = 0.8:1. Moreover, the EDX confirmed the presence of both Tb^3+^ and Eu^3+^ ions, whereas the latter is in low amount (Tb^3+^/Eu^3+^ ratio = 17.5). Therefore, taking into account the aforementioned facts, as well as the presence of Cl^−^, we can assume that the majority of *acac* moieties have been coordinated to Ln^3+^ ions, being the first coordination sphere presumably completed by chlorides and water molecules.

IR spectra of nanoparticles synthesized in all steps are shown in Figure S4, Supporting Information. The IR spectrum of sample IONP@SiO_2_ clearly indicates the formation of the silica shell through the appearance of the conventional stretching vibrations ν(Si-O-Si) and ν(Si-OH) in the 700–1400 cm^−1^ spectral window. Moreover, the bands assigned to the free and coordinated to Ln^3+^ *acac* ligand may be observed on the IR spectra of samples IONP@SiO_2_ and IONP@SiO_2_-*acac*/Tb^3+^/Eu^3+^, respectively (Figure S4, Table S2, SI). However, due to the low intensity of the characteristic bands of the *acac* ligand and their overlapping with the bending vibrations of the OH groups from water, it was not possible to clearly state on the coordination of the lanthanide ions to the *acac* ligand in IONP@SiO_2_-*acac*/Tb^3+^/Eu^3+^.

TEM images of samples at different stages of preparation demonstrate that each single IONP core is surrounded by a clearly determined silica shell in IONP@SiO_2_, IONP@SiO_2_-*acac*, and IONP@SiO_2_-*acac*/Tb^3+^/Eu^3+^ (Figure 2b,c,d, Figure 1). The size distribution for the IONP@SiO_2_-*acac*/Tb^3+^/Eu^3+^ sample indicates an IONP core size of 26.0 ± 1.8 nm and a silica shell thickness of 11.2 ± 0.3 nm (Figure S5, SI). Note that the sizes and the shape of the IONP nanoparticles have not been altered by the silica coating, ligand grafting, and the coordination of Ln^3+^ (Figure 2, Figures S1 and S5, SI). Moreover, quasi monodispersed nanoparticles have been obtained in all steps (the standard deviations (SD) for all samples are less than 5% except one, for which it is less than 7%, see Figure 2c).

The topochemical distribution visualized by HAADF-STEM with the EDX mapping confirms the homogeneous distribution of Si and Tb^3+^ on the surface of IONP@SiO_2_-*acac*/Tb^3+^/Eu^3+^ nanoparticles, whereas Eu^3+^ could not be detected because of its very low amount (Figure 1d). This situation has already been seen in another work [9]. The DLS results (Figures S1c and S6, Supporting Information) indicate that the nanoparticles obtained in all steps are not aggregated.

### 3.2. Magnetic Characterization and Heating Property

**Figure 3:**
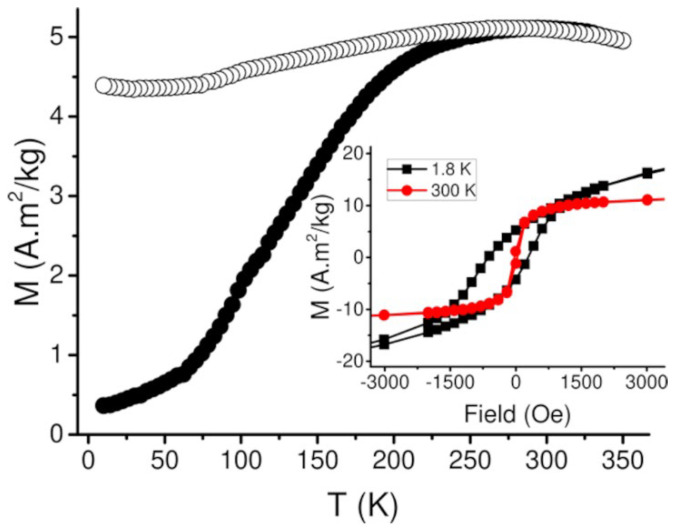
ZFC-FC curves for IONP@SiO_2_-*acac*/Tb^3+^/Eu^3+^ measured under an applied magnetic field of 100 Oe. Inset: Variation of the magnetization as a function of magnetic field of IONP@SiO_2_-*acac*/Tb^3+^/Eu^3+^ at 1.8 K (black square) and 300 K (red circle) shown between −3 and 3 kOe.

The magnetic behavior of the IONP@SiO_2_-*acac*/Tb^3+^/Eu^3+^ nanoparticles was investigated in powder by using a SQUID-MPMS magnetometer working in the 1.8–350 K temperature range up to 5 T. A classical profile of the temperature dependence of the magnetization performed in the Zero Field Cooled (ZFC)/Filed Cooled (FC) modes under an applied field of 100 Oe with *T_max_* = 294 K can be observed in Figure 3. One can note the flatness (even decrease) of the FC curve as the temperature decreases, which can be interpreted as a hallmark of the superspin glass-like state at low temperature for the magnetic nanoparticles, rather than a superparamagnetic behavior [52].The field dependences of the magnetization at 1.8 K (black squares) and at 300 K (red circles) (Inset of Figure 3, Figure S7, SI) show the open hysteresis loop at low temperature with the coercive field of 908 Oe (at 1.8 K), whereas hysteresis is closed at 300 K. This static behavior is coherent with the previously reported results on IONPs [53].

The dynamic magnetic measurements were investigated using AC mode with different frequencies to determine the magnetic regime of these nanoparticles. The temperature dependence of the in-phase, χ′, and the out-of-phase, χ″, components of the AC susceptibility performed with frequencies ranging from 5 to 1201.9 Hz in a zero static field and with the oscillating field of 3 Oe shows a series of frequency dependent images, shown in Figure S8, SI. At 5 Hz, both, χ′ and χ″ responses present a maximum at 255 and 223 K, respectively, which shift toward higher temperatures as the frequency increases. This temperature dependence of the AC susceptibility is specific to the short-range magnetic ordering of nanoparticles. The temperature dependence of the relaxation time, *τ*, extracted from the maximum of the χ″ component is shown in Figure S9, SI. The *τ* vs. *1/T* curve exhibits the occurrence of a rather complex dynamic behavior with the presence of low and high temperatures domains, which can be attributed to the presence of two magnetic regimes in these nanoparticles. The low temperature domain has been fitted with the Néel relaxation model (Figure S10, SI), which relates the blocking temperature with the relaxation time, *τ* = *τ*_0_*exp*(*E_a_/k_B_T_B_*), where *E_a_ = KV* is the energy barrier and *τ*_0_ is the attempt time [54]. The best fit parameters gave the value of the energy barrier of 7853 ± 308 cm^−1^ and τ_0_ = 10^−23.3 ± 0.8^ s. Such a low value of *τ_0_* is out of the 10^−9^–10^−12^ s^−1^ range usually observed for the pure superparamagnetic regime and has no physical meaning indicating that the Néel model is not appropriate to describe the dynamic of this system. However, such *τ_0_* values rather suggest the presence of a superspin glass-like behavior with magnetic non-linearities and critical dynamic scaling below the freezing temperature [52]. Its origin may usually be induced by the presence of strong dipolar interactions and/or a spin frustration on the surface of the nanoparticles or in their volume [55]. Note that such low-temperature behavior is coherent with previously published results on IONP surrounded by organic molecules [53] or coated by a mesostructured silica shell [56]. For this reason, we verified whether the dynamic of the relaxation time in low temperature regime would exhibit critical reduction, as observed in canonical spin glasses or superspin glass-like nanoparticles. The frequency-dependent relaxation time has been fitted by the Vogel–Fulcher law, *τ* = *τ*_0_*exp*(*E_a_/kB*(*T − T*_0_)), where *T*_0_ represents an additional parameter taking into account dipolar interactions between nanoparticles [36]. The obtained parameters (*E_a_* = 7853 ± 345 cm^−1^, *τ*_0_ = 10^−23.3 ± 0.9^ s, and *T*_0_ = 5 × 10^−11^ ± 1 × 10^−6^ K) indicated that *T*_0_ is very small and *τ*_0_ is not larger than the one obtained by using the Néel model (Figure S10, SI), suggesting that, in our system, the strength of the magnetostatic interactions is very low. This behavior is different in comparison to what we observed in the case of similar IONPs surrounded by organic molecules [53] and signify that the observed complex superspin glass-like behavior is induced by the presence of a complex interface with a silica shell leading to the surface and interface spin frustration [36]. Note that the presence of lanthanide ions did not importantly impact the magnetic behavior of the nanoparticles.

**Figure 4:**
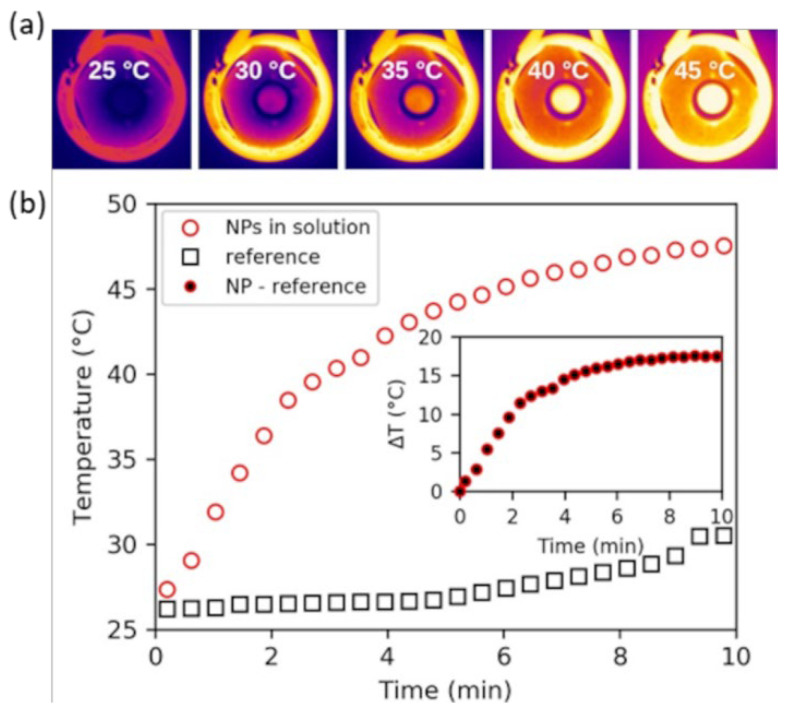
(**a**) Thermal images of a vial containing the IONP@SiO_2_-*acac*-Ln^3+^ solution in the presence of the AC magnetic field (350 kHz, 20 mT). (**b**) Temperature measurements as a function of time of IONP@SiO_2_-*acac*/Ln^3+^ solution (red line) and ethanol (black line) subjected to an AC magnetic field. Inset: temporal evolution of the change in temperature (ΔT) calculated as the temperature difference between NPs in solution and reference.

The heating capacity of IONP@SiO_2_-*acac*/Tb^3+^/Eu^3+^ was evaluated by measuring the temperature of ethanolic solutions (16 mg·mL^−1^) under an ac magnetic field (≈20 mT at a frequency of 350 kHz) by a thermal camera (Figure 4a). A rapid temperature rise up to approximately 48 °C suitable for a minimally invasive hyperthermia treatment [57] was observed in the 10 min of exposure (Figure 4b, red circles). In the absence of the nanoparticles (Figure 4b, black squares), the temperature increment is clearly lower (28 °C after 10 min of exposure). Although it is difficult to directly compare the heating capacity of IONP/OA/OA and IONP@SiO_2_-*acac*/Tb^3+^/Eu^3+^ because the measurements were done in different solvents, the temperature elevation at the macroscopic level for the latter is, however, strongly impacted by the silica shell (Figure S11, SI).

### 3.3. Optical Characterization, Temperature Sensing, and Theoretical Modelling

**Figure 5:**
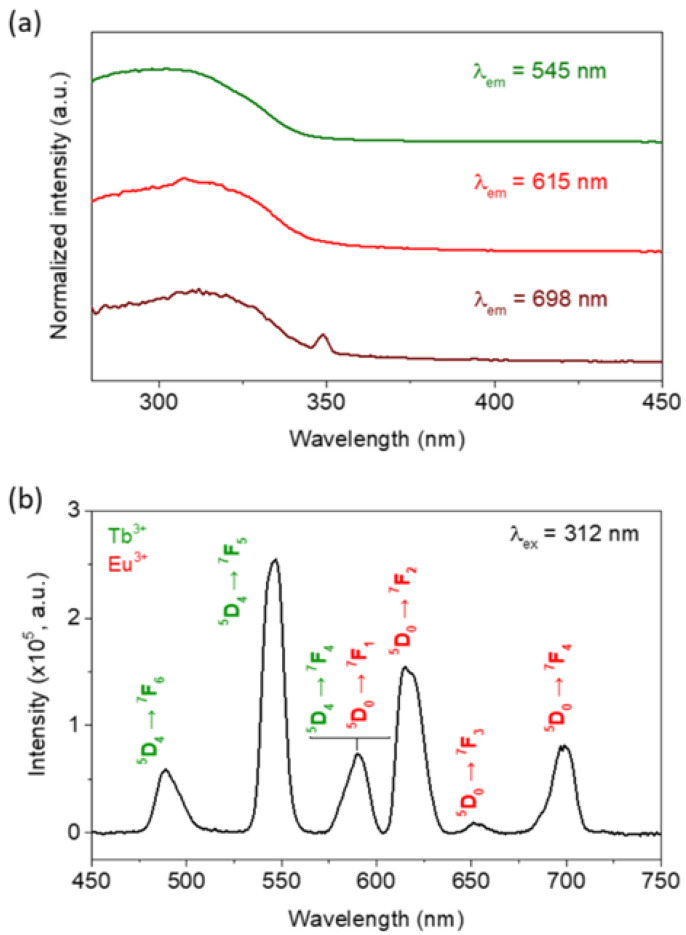
(**a**) Room temperature excitation spectra of IONP@SiO_2_-*acac*/Tb^3+^/Eu^3+^ monitored at λ_em_ = 545, 615, and 698 nm. (**b**) Room temperature emission spectrum performed with λ_ex_ = 312 nm for IONP@SiO_2_-*acac*/Tb^3+^/Eu^3+^. The Eu^3+^ and Tb^3+^ based transitions are written in red and green, respectively.

**Figure 6:**
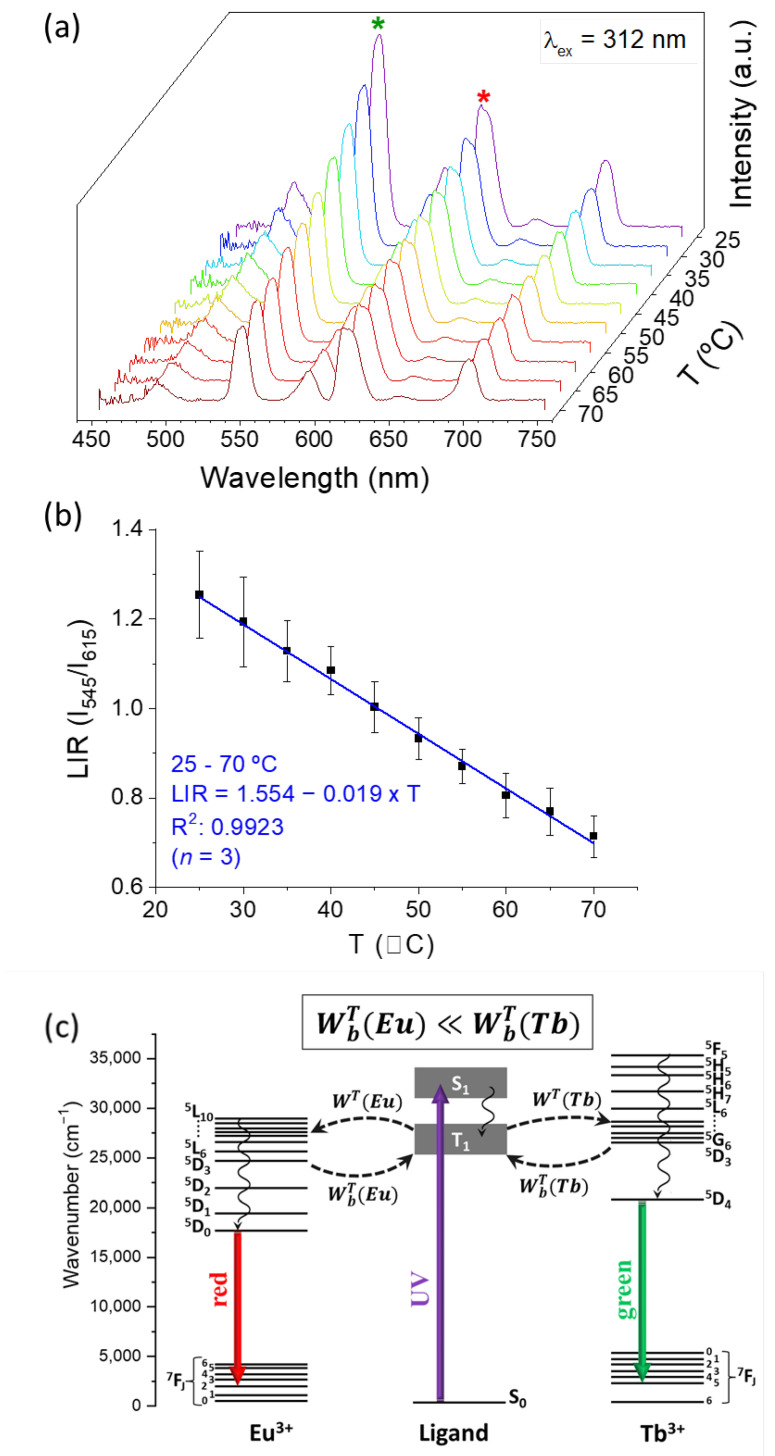
(**a**) Emission spectra of IONP@SiO_2_-*acac*-Ln^3+^ (λ_ex_ = 312 nm) recorded at temperatures between 25 and 70 °C. The green and red asterisk symbols indicate the Tb^3+^: ^5^D_4_ → ^7^F_5_ (530–565 nm) and Eu^3+^: ^5^D_0_ → ^7^F_2_ (603–637 nm) transitions, of which integrated areas were used to construct (**b**) the calibration curve LIR vs. temperature. The solid line represents a linear fitting. The error bars correspond to standard error of mean determined from three consecutive temperature cycles. (**c**) Simplified energy level diagram showing the intra-4f transitions observed in Figure 5b and the effect of the increase of the backward IET rates $W_{b}^{T}$ with the temperature; see SI for further details on the IET calculations.

The luminescence properties of IONP@SiO_2_-*acac*/Tb^3+^/Eu^3+^ were first investigated at room temperature. The excitation spectrum (Figure 5a and Figure S12, SI) measured by monitoring the Tb^3+^ main emission at 545 nm (^5^D_4_ → ^7^F_5_) shows a broad band with a maximum at 312 nm. This band accounts for the so-called antenna effect caused by the presence of the *acac* ligands and confirms their coordination to the lanthanide ions [58]. Note that the action of the β-diketones (such as *acac*) as antennas due to the high efficiency of energy transfer to Ln^3+^ is well known in the literature [59]. The emission spectrum (Figure 5b) recorded under excitation at 312 nm displays peaks at 489, 545, 590, 615, 651, and 699 nm, which are assigned to the characteristic intra-4*f* transitions arising from Tb^3+ 5^D_4_ to the ^7^F_J_ manifold (J = 6, 5, 4) [60] and Eu^3+ 5^D_0_ to the ^7^F_J_ manifold (J = 1, 2, 3, 4) [61]. The corresponding Dieke’s diagram is shown in Figure 6. The Tb^3+^ green emission (545 nm, ^5^D_4_ → ^7^F_5_) dominates the general intensity, followed by the Eu^3+^ red one (615 nm, ^5^D_0_ → ^7^F_2_). The excitation spectra recorded by monitoring the Eu^3+^ emission at 615 nm (^5^D_0_ → ^7^F_4_) and 698 nm (^5^D_0_ → ^7^F_4_) (Figure 5a) do not display any Tb^3+^ transitions precluding the occurrence of Tb^3+^-to-Eu^3+^ energy transfer. This is expected due to a random distribution of Ln^3+^ ions at the IONP surface, implying large Tb^3+^–Eu^3+^ distances [62,63]. The most plausible energy transfer mechanism is, thus, due to the intramolecular energy transfer (IET) from donor (triplet excited state of the ligand, T_1_) to acceptors Ln^3+^ levels, as depicted in Figure 6c.

Although SiO_2_-*acac* has a larger molecular framework than the precursor *acac*, both have conjugated systems located around the Ln^3+^ ion, and it is expected that the T_1_ states of the SiO_2_-*acac* do not shift greatly towards into the short wavelength region, in contrast to the T_1_ of the *acac* (T_1_ with energy lying above 25,500 cm^−1^) [37,64,65]. Therefore, we assumed a consistent value of T_1_ = 27,000 cm^−1^ for the SiO_2_-*acac* in the IET calculations. Thus, the IET rates from the ligand to Ln^3+^ ions were calculated according to Equations (3)–(5) [38,39,40,66]. The most relevant forward rates for Tb^3+^ are the T_1_ → [^7^F_6_ → ^5^G_6_] and T_1_ → [^7^F_5_ → ^5^G_5_], which together represent 90% of the total $W^{T}$, whereas the backward is predominantly composed (~85% of the total $W_{b}^{T}$ rate) of four transitions from [^5^G_6_ → ^7^F_6_], [^5^G_5_ → ^7^F_6_], [^5^H_5_ → ^7^F_5_], and [^5^F_5_ → ^7^F_5_] → T_1_. However, the thermal effect involves the ^5^G_6_ and ^5^G_5_ levels due to their energy resonant condition with the T_1_ state (δ in the order of k_B_T); see pathways 3 and 5 in Table S4. However, the Eu^3+^ presented only the [^7^F_1_↔^5^G_2_] transition as the most important one for both forward (~85%) and backward rates (~99%); see pathway 13 in Table S3. Hence, once the ^7^F_1_ is involved, the thermal behavior of the Eu^3+^ is a direct consequence of the increase in the ^7^F_1_ population when temperature rises [62,67].

**Figure 7:**
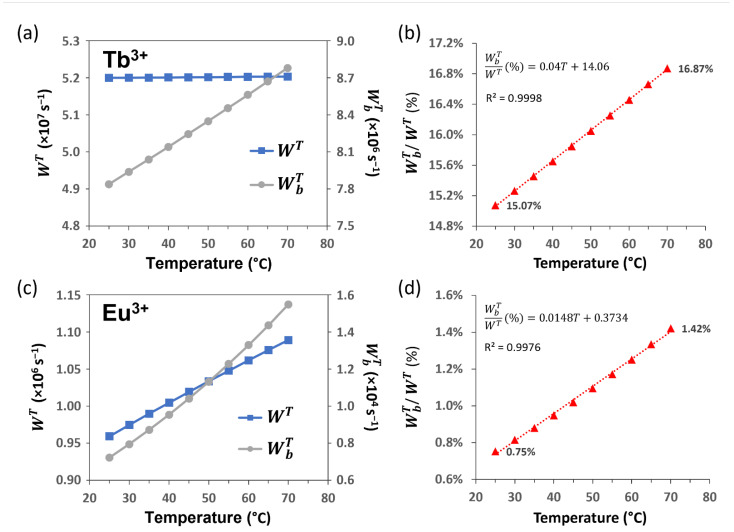
IET rates involving the ligand T_1_ state. Forward $W^{T}$ and backward $W_{b}^{T}$ for (**a**) Tb^3+^ and (**c**) Eu^3+^ complexes (in s^−1^) as a function of the temperature; (**b**,**d**) show the growing behavior of the $W_{b}^{T}$ regarding the $W^{T}$ with temperature.

Our theoretical analysis showed that the total IET rates $W^{T}$ (forward rates, ligand-to-Ln^3+^, Figure 6c) is constant for the Tb^3+^, whereas it increases for the Eu^3+^ when the temperature rises (Figure 7). All back transfer rates $W_{b}^{T}$ (Ln^3+^-to-ligand, Figure 6c) increase with temperature, which may favor the emission intensities decreasing of the Eu^3+^ and Tb^3+^ ions, as observed in Figure 6a. However, the Tb^3+^ is more sensitive due to its relatively high $W_{b}^{T}$, which represents a reasonable percentage of the forward $W^{T}$ (Figure 7b), reflecting a faster depopulation of the Tb^3+ 5^D_4_ than the Eu^3+ 5^D_0_ level when temperature increases.

In order to verify if our nanoparticles can be used as an emissive thermometer, the emission spectra of IONP@SiO_2_-*acac*/Tb^3+^/Eu^3+^ were measured in the 25–70 °C temperature range under excitation at 312 nm (Figure 6a). The luminescence intensity ratio (LIR) between Tb^3+^ and Eu^3+^ main emissions (at 545 nm and 615 nm, respectively) shows a linear temperature dependence (Figure 6b), which enables the use of these nanoparticles as a self-referencing temperature sensor. The error bars represent the standard deviation of average values obtained upon three consecutive temperature cycles (Figure S14, SI). Note also that the absence of the Ln^3+^ ions leaching has been confirmed by taking the emission spectrum of remaining solution after removal of the nanoparticles by centrifugation after the first heating cycle. This emission spectrum free of nanoparticles did not present Ln^3+^ ions characteristic peaks.

The photo-stability was investigated by monitoring the emission spectra at room temperature after different periods of exposure to UV light (Figure S14, SI). The emission intensity was affected by UV-light irradiation with an estimated photo-degradation rate (i.e., emission intensity decrease per time) of less than 0.5% per minute for both, Tb^3+^ green and Eu^3+^ red transitions. Despite the photo-degradation effect resulting in the intensity decrease between cycles (Figure S14d, Supporting Information), the ratiometric feature of the sensing method offers the advantage of taking this variation into account in the thermometric parameter (LIR).

The calibration parameters and metrics related to the thermometric performance are provided in Table 1. The regression coefficient (*R^2^*) revealed excellent calibration linearity (*R^2^* = 0.9923) in the operating temperature range 25–70 °C. The repeatability represents the variability among the measurements and is determined from the maximum relative standard deviation (RSD) [68]. The highest RSD observed among several heating cycles was 8.44%.

The relative thermal sensitivity (*S_r_*) is the parameter used to describe and compare the sensing performance of different types of thermometers [67]. The *S_r_* refers to the relative variation rate of the thermometric parameter (LIR in the present system) per degree of temperature, expressed as:

(12) $$S_{r}\left(T\right)=\left|\partial LIR\left(T\right)/\partial T\right|/LIR\left(T\right)\times 100\%.$$

The highest *S_r_* value observed was 1.64%·K^−1^ at 40 °C, which is satisfactory considering that high relative thermal sensitivities are frequently considered to be around 1%·K^−1^ [68]. Luminescent thermometers based on Tb^3+^/Eu^3+^-complexes covalently grafted to mesoporous silica using dipyridyl–pyridazine ligands presented the highest *S_r_* of 1.32%·K^−1^ in the cryogenic range (−13 °C) and around 1.2%·K^−1^ in the physiological range [69].

Temperature uncertainty (or thermal resolution, *δT*) is the smallest temperature change that can be detected [68]. The *δT* value is correlated to *S_r_* as follows:

(13) $$\delta T=\left|\delta LIR\left(T\right)/LIR\left(T\right)\right|/S_{r}\left(T\right),$$

where *δ*LIR(T) is the standard deviation of LIR(T) determined from multiple heating cycles. According with this, the minimal thermal resolution found for the present system is 0.03 °C. This value is adequate considering the narrow temperature working range (41.8–45 °C) of hyperthermia [57], which demands high thermal resolution (≤0.1 °C) [70].

The thermometric performance of mixed Tb^3+^/Eu^3+^ compounds were recently summarized by Brites et al. [68]. The Tb^3+^/Eu^3+^-based thermometers are mostly molecular complexes or organic–inorganic hybrids presenting downshifted visible emission under UV-visible excitation. In these systems, the thermometric methods are based on energy transfer mechanisms between Tb^3+^ and Eu^3+^, as well as between Ln^3+^ and antenna ligands or host. In this sense, the temperature sensitivity of Tb^3+^ and Eu^3+^ emissions can be adjusted by varying the ligands [71] or the Tb^3+^/Eu^3+^ ratio [62]. The emissive level of Tb^3+^ (^5^D_4_) is energetically higher than the Eu^3+^ one (^5^D_0_), which favors its thermally driven depopulation caused by Tb^3+^-to-ligand energy transfer (Figure 6c) [9]. The higher thermal sensitivity of the Tb^3+^ green emission intensity compared to the Eu^3+^ red one enables the elaboration of ratiometric methods, in which the Eu^3+^ red emission works as a reference signal.

## 4. Conclusions

In summary, multifunctional magneto-luminescent core@shell IONP@SiO_2_ nanoparticles with covalently attached to the silica surface Tb^3+^/Eu^3+^(*acac*) moieties were synthesized and characterized. These nanoparticles present the clearly distinguished single IONP core of ca. 26 nm and the well-defined silica shell with controlled thickness. This system exhibits the Tb^3+^ and Eu^3+^ characteristic visible emissions and suitable magnetic properties that make it efficient as a nanoheater with a Ln^3+^-based emissive temperature sensor covalently coupled to it. The experimental investigations of photo-luminescence in solution coupled with theoretical modelling demonstrated the occurrence of an intramolecular energy transfer from donor (triplet excited state of the ligand, T_1_) to acceptors Ln^3+^ levels. The static and dynamic investigations of the magnetic behavior revealed that the nanoparticles exhibit a complex superspin glass-like behavior induced by surface and/or interface spin frustration that occurred in each nanoparticle, and the magnetostatic interactions do not importantly impact the magnetic regime. Note that this behavior is different in comparison with other similar IONPs with attached organic molecules on their surface or coated with mesostructured silica shell, for which the presence of dipolar interactions plays an important role in the appearance of a spin glass-like comportment. The freezing temperature occurred near room temperature, which permits the rapid heating of their solution up to 48 °C for 10 min under an applied ac magnetic field. In addition, the thermometer proposed here operates in a broad temperature range relevant for magnetothermia-related applications, with good photostability and reproducibility after multiple heating cycles. The possibility to design multifunctional magneto-luminescent nano-systems with a controlled morphology of a single nanoheater enwrapped by a thin silica shell containing an emissive thermometer opens new perspectives for accurate temperature detection during magnetic liquid hyperthermia therapy and represents the first step towards the local temperature measurements in unique magnetic nanoparticles. Given that the described Tb^3+^/Eu^3+^(*acac*) sensor should be excited in the UV domain and the emission occurs in the visible region, which is not compatible with the biological applications, the use of NIR-emissive complexes based on Nd^3+^, Er^3+^, or Yb^3+^ ions will constitute the next step of our study in the aim to design biologically relevant temperature sensors.

## Figures and Tables

**Table 1:** Calibration parameters of the thermometer IONP@SiO_2_-*acac*/Tb^3+^/Eu^3+^.

Parameter	Value
Temperature operating range	25–70 °C
Slope	−1.22 × 10^−2^ (±3.60 × 10^−4^)
Intercept	1.55 (±1.94 × 10^−2^)
Regression coefficient (*R*^2^)	0.9923
Relative thermal sensitivity (S_r_)	1.64%·K^−1^ at 40 °C
Uncertainty (*δT*)	0.03 °C
Repeatability (*RSD*)	8.44%

## Data Availability

The data presented in this study are available on request from the corresponding authors.

## Supplementary Materials

The following supporting information can be downloaded at: https://www.mdpi.com/article/10.3390/nano12183109/s1. Figure S1: (a) TEM image of IONP/OA/OA and (b) corresponding particle size distribution (*n* = 100); (c) dynamic size distribution of IONP/OA/OA solution in cyclohexane estimated by DLS; (d) XRD pattern of IONP/OA/OA powder and reference diffraction peaks corresponding to Fe_3_O_4_ and FeO; Figure S2: TGA analyses obtained with a 5 °C min^−1^ heating rate under air for: (a) IONP@SiO_2_ (*ca*. 11 nm-shell) and (b) IONP@SiO_2_-*acac*; Figure S3: BET adsorption/desorption isotherms for nitrogen adsorption capacity of IONP@SiO_2_. Specific surface of 315 m^2^·g^−1^ has been estimated by Brunauer−Emmett−Teller (BET) method; Figure S4: IR spectra of IONP/OA/OA, IONP@SiO_2_, IONP@SiO_2_-*acac*/Ln^3+^, and *acac*-silane in: (a) the full analyzed range 4000–400 cm^−1^, (b) the magnification in the window 1800–1400 cm^−1^, (c) the magnification in the window 600–400 cm^−1^; Figure S5: Size distributions of (a) IONP@SiO_2_-*acac* and (b) IONP@SiO_2_-*acac*/Tb^3+^/Eu^3+^ (number of counted particles = 100); Figure S6: DLS size distribution of IONP@SiO_2_ (*ca*. 11 nm-shell), IONP@SiO_2_-*acac*, and IONP@SiO_2_-*acac*/Ln^3+^ dispersed in ethanol with average dynamic diameters of 159, 150, and 151 nm, respectively; Figure S7: Field dependence of the magnetization for IONP@SiO_2_-*acac*/Tb^3+^/Eu^3+^ nanoparticles at 1.8 K (black square), 10 K (blue diamond), and 300 K (red circle); Figure S8: Temperature dependence of: (a) in-phase, χ′, and (b) out-of-phase, χ″, components of the AC magnetic susceptibility performed at different frequencies for IONP@SiO_2_-*acac*/Tb^3+^/Eu^3+^ nanoparticles; Figure S9: Relaxation time vs. *1/T* curve extracted from the frequency dependence of the out-of-phase component of the magnetic susceptibility; Figure S10: Low temperature region of relaxation time vs. *1/T* curve extracted from the frequency dependence of the out-of-phase component of the AC magnetic susceptibility (black circle) and its theoretical fit by using the Arrhenius model (red line) and the Vogel–Fulcher model (blue dashed line) for IONP@SiO_2_-*acac*/Tb^3+^/Eu^3+^ nanoparticles; Figure S11: Temperature measurements as a function of time of IONP/OA/OA in cyclohexane (red) and IONP@SiO_2_-acac/Ln^3+^ solution in ethanol (blue) and reference (black) subjected to an AC magnetic field (**≈** 20 mT at a frequency of 350 kHz); Figure S12: High-resolution spectrum of excitation spectrum monitored at λ_em_ = 698 nm. The peak at 348 nm is the half-order of the monitored wavelength; Figure S13: (a) Emission spectra (λ_ex_ = 312 nm) at different temperatures for IONP@SiO_2_-*acac*/Tb^3+^/Eu^3+^ obtained upon three consecutive temperature cycles; (b) their respective normalized integrated intensities; and (c) LIR between 545 nm and 615 nm. Wavelength ranges for integrating areas: 530–565 nm (Tb^3+^:^5^D_4_ → ^7^F_5_) and 603–637 nm (Eu^3+^: ^5^D_0_ → ^7^F_2_). (d) Relative intensity of green and red emissions at 25 °C along the heating cycles; Figure S14: (a) Emission spectra of IONP@SiO_2_-*acac*/Ln^3+^ measured after different periods of exposure to excitation light (λ_ex_ = 312 nm) and (b) relative intensities at 545 nm and 615 nm normalized to the spectrum measured at time t = 0. Wavelength ranges for integrating areas: 530–565 nm (Tb^3+^: ^5^D_4_ → ^7^F_5_) and 603–637 nm (Eu^3+^:^5^D_0_ → ^7^F_2_); Table S1: Size distribution of IONP@SiO_2_ core@shell nanoparticles and related amount of TEOS; Table S2: Band assignment for IR spectra of IONP/OA/OA, IONP@SiO_2_, IONP@SiO_2_-acac/Ln^3+^, and acac-silane; Table S3: IET rates (in s^−1^) from T_1_ (donor) to Eu^3+^ ion acceptor states; *δ* is the donor–acceptor energy difference (in cm^−1^); $W_{d-d}$, $W_{d-m}$, and $W_{ex}$ are the dipole–dipole, dipole–multipole, and exchange rates (in s^−1^); $W^{T}$ and $W_{b}^{T}$ are the forward and backward energy transfer rates for each pathway at 25 °C; Table S4: IET rates (in s^−1^) from T_1_ (donor) to Tb^3+^ ion acceptor states; *δ* is the donor–acceptor energy difference (in cm^−1^); $W_{d-d}$, $W_{d-m}$, and $W_{ex}$ are the dipole–dipole, dipole–multipole, and exchange rates (in s^−1^); $W^{T}$ and $W_{b}^{T}$ are the forward and backward energy transfer rates for each pathway at 25 °C.
