# Supplementary material for: Magneto-Induced Hyperthermia and Temperature Detection in Single Iron Oxide Core-Silica/Tb3+/Eu3+(Acac) Shell Nano-Objects
Source: Nanomaterials (Basel). 2022 Sep 7;12(18):3109. doi: 10.3390/nano12183109 (PMC9503042; doi:10.3390/nano12183109)
Supplement: Supplementary file 1 [file nanomaterials-12-03109-s001.zip › nanomaterials-1848761-supplementary.pdf]

Supporting Information

Magneto-induced hyperthermia and temperature detection in single iron oxide core - silica/Tb^{3+}/Eu^{3+}(*acac*) shell nano-objects

Karina Nigoghossian [1,*], Basile Bouvet [1], Gautier Félix [1], Saad Sene [1], Luca Costa [2], Pierre-Emmanuel Milhet [2], Albano N. Carneiro Neto [3], Luis D. Carlos [3], Erwan Oliviero[1], Yannick Guari [1,*] and Joulia Larionova [1,*]

[1] ICGM, Univ. Montpellier, CNRS, ENSCM, Montpellier, France
[2] Centre de Biologie Structurale (CBS), Univ. Montpellier, CNRS, INSERM, Montpellier, France
[3] Phantom-g, Physics Department and CICECO – Aveiro Institute of Materials, University of Aveiro, 3810-193, Aveiro, Portugal
* Correspondence: joulia.larionova@umontpellier.fr; karina.nigoghossian@umontpellier.fr; yannick.guari@umontpellier.fr

Contents

Tables **2**
Table S1 2
Table S2 2
Table S3 3
Table S4 4
Figures **5**
Fig. S1 5
Fig. S2 6
Fig. S3 7
Fig. S4 8
Fig. S5 8
Fig. S6 9
Fig. S7 9
Fig. S8 10
Fig. S9 11
Fig. S10 11
Fig. S11 13
Fig. S12 12
Fig. S13 13
Fig. S14 14
References **14**

Tables

Table S1. Size distribution of $IONP@SiO_2$ core@shell nanoparticles and related amount of TEOS.

	**SiO_2 shell thickness**	**Whole nanoparticle's size**	**IONP core size**	**Well organisation of SiO_2 shell around the IONP core**
1.5	8.3 ± 0.2	43.1 ± 2.3	26.6 ± 2.0	no
2	10.7 ± 0.4	46.9 ± 2.5	25.4 ± 1.6	yes
4	13.7 ± 0.5	53.1 ± 3.7	25.8 ± 2.6	yes
6	18.4 ± 0.1	62.0 ± 2.6	25.2 ± 2.3	yes
8	20.8 ± 0.1	66.6 ± 2.1	24.9 ± 1.9	yes
10	22.6 ± 0.1	71.2 ± 3.1	26.0 ± 2.9	yes
12	27.9 ± 0.4	80.8 ± 3.0	24.9 ± 2.3	yes

Table S2. Band assignment for IR spectra of IONP/OA/OA, $IONP@SiO_2$, $IONP@SiO_2$-*acac*/Ln^{3+} and *acac*-silane. [1]

**IONP/OA/OA**	**$IONP@SiO_2$**	**$IONP@SiO_2$-acac/Ln^{3+}**	**Acac-silane**	**Assigments**
3315	-	-	-	ν(N−H) from *NH_2*
3005	2980	2950	2974	ν(C−H) from *CH_3*
2920	2901	2916	2927	ν_{as}(C−H) from *CH_2*
2850	2870	2868	2885	ν_s(C−H) from *CH_2*
1633	-	-	-	ν(C=C)
-	1634	1714; 1634	1716; 1701	ν(C=O) *keto*
-	-	-	1585	ν(C=O) *enol*
1557	-	-	-	ν_{as}(COO)
1464	-	-	-	ν_s(COO)
-	1406	1454	-	δ_s(C−H) from *CH_2*
-	1392	1376	-	ν(C−C)
-	957	971	955	ν(Si−OH)
-	1066; 795; 456	1076; 798; 465	1073; 775; 479	ν(Si−O−Si)
720 - 568	568	585	-	ν(Fe−O)
-	-	410	-	ν(Ln−O)

Table S3. IET rates (in s^{-1}) from T_1 (donor) to Eu^{3+} ion acceptor states. δ is the donor-acceptor energy difference (in cm^{-1}). W_{d-d}, W_{d-m}, and W_{ex} are the dipole-dipole, dipole-multipole, and exchange rates (in s^{-1}). W^T and W_b^T are the forward and backward energy transfer rates for each pathway at 25 °C.

**Pathway label *(n)***	**Acceptor**	$\boldsymbol{\delta}$	$\boldsymbol{W_{d-d}}$	$\boldsymbol{W_{d-m}}$	$\boldsymbol{W_{ex}}$	$\boldsymbol{W^T}$ **(forward)**	$\boldsymbol{W_b^T}$ **(backward)**
1	$^7F_0 \rightarrow {}^5D_0$	9707	3.61×10^{-2}	1.93×10^{1}	0	3.25×10^{-2}	1.47×10^{-22}
2	$^7F_0 \rightarrow {}^5D_1$	7973	0	0	1.35×10^{5}	9.05×10^{4}	1.77×10^{-12}
3	$^7F_0 \rightarrow {}^5D_2$	5517	1.57	8.39×10^{2}	0	5.65×10^{2}	1.55×10^{-9}
4	$^7F_0 \rightarrow {}^5L_6$	1675	2.52×10^{2}	4.91	0	1.73×10^{2}	5.33×10^{-2}
5	$^7F_0 \rightarrow {}^5G_6$	248	7.53×10^{1}	1.47	0	5.16×10^{1}	1.56×10^{1}
6	$^7F_0 \rightarrow {}^5D_4$	-586	2.19×10^{1}	3.56×10^{1}	0	2.28	3.86×10^{1}
7	$^7F_1 \rightarrow {}^5D_0$	10079	0	0	1.02×10^{4}	3.33×10^{3}	2.51×10^{-18}
8	$^7F_1 \rightarrow {}^5D_1$	8345	7.98×10^{-2}	4.27×10^{1}	2.89×10^{1}	2.35×10^{1}	7.62×10^{-17}
9	$^7F_1 \rightarrow {}^5D_2$	5889	0	0	1.66×10^{5}	5.43×10^{4}	2.47×10^{-8}
10	$^7F_1 \rightarrow {}^5D_3$	3017	5.41	7.30×10^{2}	0	2.41×10^{2}	1.15×10^{-4}
11	$^7F_1 \rightarrow {}^5L_6$	2047	4.49×10^{1}	8.74×10^{-1}	0	1.50×10^{1}	7.70×10^{-4}
12	$^7F_1 \rightarrow {}^5L_7$	1015	1.14×10^{2}	2.22	0	3.81×10^{1}	2.84×10^{-1}
13	$^7F_1 \rightarrow {}^5G_2$	980	0	0	2.47×10^{6}	8.10×10^{5}	7.16×10^{3}
14	$^7F_1 \rightarrow {}^5G_3$	750	9.14	7.11×10^{2}	0	2.36×10^{2}	6.33
15	$^7F_1 \rightarrow {}^5G_6$	620	3.24×10^{1}	6.31×10^{-1}	0	1.08×10^{1}	5.44×10^{-1}
16	$^7F_1 \rightarrow {}^5G_5$	609	6.69×10^{1}	5.55	0	2.38×10^{1}	1.26
					$\boldsymbol{W^T}$	$\mathbf{9.59\times10^{5}}$	
					$\boldsymbol{W_b^T}$		$\mathbf{7.22\times10^{3}}$

Note: The IET involving the transition $^7F_0 \rightarrow {}^5D_0$ was calculated considering the J-mixing effect.

Table S4. IET rates (in s^{-1}) from T_1 (donor) to Tb^{3+} ion acceptor states. δ is the donor-acceptor energy difference (in cm^{-1}). W_{d-d}, W_{d-m}, and W_{ex} are the dipole-dipole, dipole-multipole, and exchange rates (in s^{-1}). W^T and W_b^T are the forward and backward energy transfer rates for each pathway at 25 °C.

**pathway label *(n)***	**acceptor**	$\boldsymbol{\delta}$	$\boldsymbol{W_{d-d}}$	$\boldsymbol{W_{d-m}}$	$\boldsymbol{W_{ex}}$	$\boldsymbol{W^T}$ **(forward)**	$\boldsymbol{W_b^T}$ **(backward)**
1	$^7F_6 \rightarrow ^5D_4$	6556	2.06×10^{-1}	2.71×10^{1}	0	2.73×10^{1}	4.98×10^{-13}
2	$^7F_6 \rightarrow ^5D_3$	764	2.88	3.55×10^{-1}	0	3.23	8.10×10^{-2}
3	$^7F_6 \rightarrow ^5G_6$	577	2.24×10^{2}	1.30×10^{3}	3.19×10^{7}	3.19×10^{7}	1.97×10^{6}
4	$^7F_6 \rightarrow ^5L_{10}$	29	1.10×10^{2}	1.56	0	1.11×10^{2}	9.67×10^{1}
5	$^7F_6 \rightarrow ^5G_5$	-767	2.89×10^{1}	9.49×10^{2}	2.15×10^{6}	5.31×10^{4}	2.15×10^{6}
6	$^7F_6 \rightarrow ^5D_2$	-1107	1.37	1.31×10^{-2}	0	6.62×10^{-3}	1.38
7	$^7F_6 \rightarrow ^5G_4$	-1287	1.51×10^{1}	7.32×10^{1}	0	1.77×10^{-1}	8.82×10^{1}
8	$^7F_6 \rightarrow ^5L_9$	-1408	7.93×10^{1}	3.85	0	9.31×10^{-2}	8.31×10^{1}
9	$^7F_6 \rightarrow ^5G_3$	-1977	4.45	1.69×10^{-1}	0	3.32×10^{-4}	4.62
10	$^7F_6 \rightarrow ^5L_8$	-2190	3.08×10^{1}	4.13×10^{-1}	0	8.03×10^{-4}	3.12×10^{1}
11	$^7F_6 \rightarrow ^5L_7$	-2457	1.50×10^{1}	3.63×10^{2}	1.45×10^{1}	2.78×10^{-3}	3.92×10^{2}
12	$^7F_6 \rightarrow ^5G_2$	-2531	5.74×10^{-1}	5.49×10^{-3}	0	2.88×10^{-6}	5.80×10^{-1}
13	$^7F_6 \rightarrow ^5L_6$	-2610	0	0	3.13×10^{5}	1.06	3.13×10^{5}
14	$^7F_6 \rightarrow ^5H_7$	-4379	0	0	1.35×10^{5}	8.94×10^{-5}	1.35×10^{5}
15	$^7F_6 \rightarrow ^5H_6$	-5891	0	0	3.28×10^{5}	1.48×10^{-7}	3.28×10^{5}
16	$^7F_6 \rightarrow ^5H_5$	-6767	0	0	1.54×10^{-3}	1.01×10^{-17}	1.54×10^{-3}
17	$^7F_6 \rightarrow ^5F_5$	-7934	0	0	7.64×10^{4}	1.80×10^{-12}	7.64×10^{4}
18	$^7F_5 \rightarrow ^5D_4$	8604	1.31×10^{-1}	4.62×10^{1}	1.19×10^{4}	1.20×10^{4}	1.11×10^{-14}
19	$^7F_5 \rightarrow ^5D_3$	2812	5.92	2.68×10^{2}	0	2.74×10^{2}	3.50×10^{-4}
20	$^7F_5 \rightarrow ^5G_6$	2625	2.10×10^{1}	2.30×10^{3}	2.99×10^{6}	2.99×10^{6}	9.44
21	$^7F_5 \rightarrow ^5L_{10}$	2077	2.39	2.29×10^{-2}	0	2.41	1.07×10^{-4}
22	$^7F_5 \rightarrow ^5G_5$	1281	1.33×10^{1}	8.64×10^{1}	1.49×10^{7}	1.49×10^{7}	3.09×10^{4}
23	$^7F_5 \rightarrow ^5D_2$	941	6.85	5.17	0	1.20×10^{1}	1.28×10^{-1}
24	$^7F_5 \rightarrow ^5G_4$	761	5.53	4.66×10^{2}	2.06×10^{6}	2.06×10^{6}	5.22×10^{4}
25	$^7F_5 \rightarrow ^5L_9$	640	3.08×10^{1}	1.67	0	3.25×10^{1}	1.48
26	$^7F_5 \rightarrow ^5L_8$	-142	5.08×10^{1}	4.33	0	2.78×10^{1}	5.51×10^{1}
27	$^7F_5 \rightarrow ^5L_7$	-409	1.56×10^{1}	1.93×10^{2}	0	2.90×10^{1}	2.09×10^{2}
28	$^7F_5 \rightarrow ^5L_6$	-562	0	0	8.31×10^{4}	5.52×10^{3}	8.31×10^{4}
29	$^7F_5 \rightarrow ^5H_6$	-3843	0	0	2.33×10^{5}	2.05×10^{-3}	2.33×10^{5}
30	$^7F_5 \rightarrow ^5H_5$	-4719	0	0	1.63×10^{6}	2.10×10^{-4}	1.63×10^{6}
31	$^7F_5 \rightarrow ^5F_5$	-5886	0	0	8.33×10^{5}	3.84×10^{-7}	8.33×10^{5}
					$\boldsymbol{W^T}$	$\boldsymbol{5.20\times10^{7}}$	
					$\boldsymbol{W_b^T}$		$\boldsymbol{7.84\times10^{6}}$

Figures

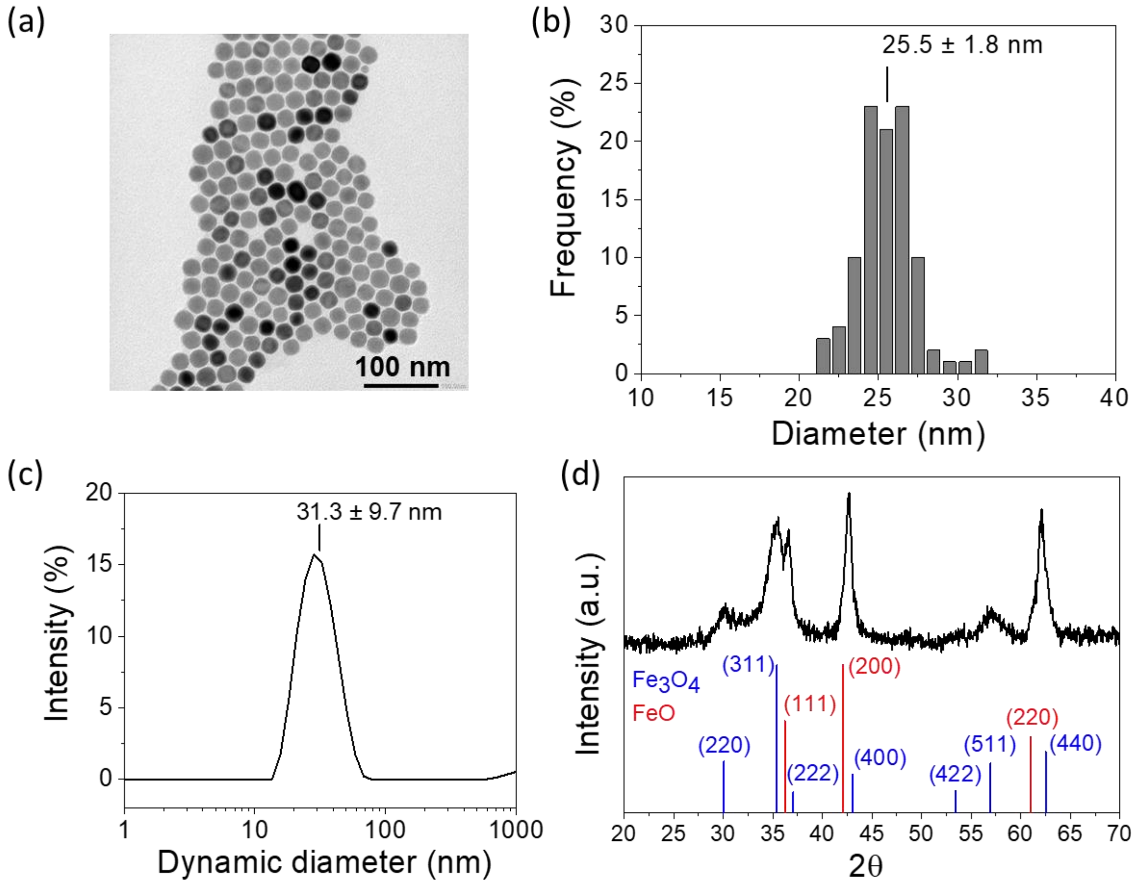

Figure S1. (a) TEM image of IONP/OA/OA and (b) corresponding particle size distribution (n = 100); (c) Dynamic size distribution of IONP/OA/OA solution in cyclohexane estimated by DLS; (d) XRD pattern of IONP/OA/OA powder and reference diffraction peaks corresponding to Fe_3O_4 [2] and FeO [3].

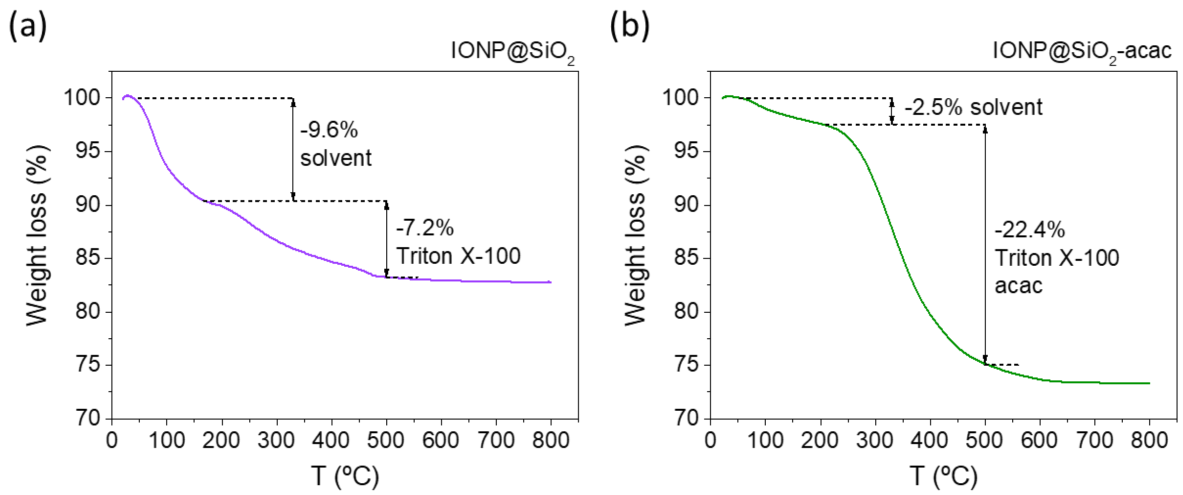

Figure S2. TGA analyses obtained with a 5 °C min^{-1} heating rate under air for: (a) IONP@SiO_2 (chosen nanoparticles with *ca*. 11 nm silica shell) and (b) corresponding IONP@SiO_2-*acac* nanoparticles. *Note:* In the thermogravimetric (TGA) curve for IONP@SiO_2, a weight loss of 9.6 % occurred at 165 °C is attributed to the solvent molecules sorbed in the silica pores. A weight loss of 7.2 % observed in the 200–450 °C temperature range can be attributed to residual Triton X-100 molecules [4] used for the micelle formation during the silica coating step [5]. In the case of IONP@SiO_2-acac, the first weight loss of 2.5% attributed to the loss of sorbed solvent molecules occurred at around 160 °C . It followed by the main loss of 22.4% between 200 and 500 °C. This latter can be associated with the thermal degradation of both, the *acac*-silane ligand grafted to the silica shell and the surfactant. The comparison between TG curves of IONP@SiO_2 and IONP@SiO_2-acac leads to the estimation of the *acac* fraction of 15.2%. This corresponds to the loss of organic moieties of *acac* (–$(CH_2)_3CH[CH_3C(O)]_2$ (141.2 g·mol^{-1})) in IONP@SiO_2-*acac* and allows the proposition of the stoichiometric formula **IONP@12.3SiO_2/$[CH_3C(O)]_2CH(CH_2)_3SiO_{1.5}$**. From SEM-EDX results (Section 2.2.5 of the main article) with the weight ratio between Lanthanides and Si (Ln^{3+}/Si = 25.8/74.2), the stoichiometric formula after Tb^{3+}/Eu^{3+} grafting was estimated to be **IONP@12.3SiO_2/ $[CH_3C(O)]_2CH(CH_2)_3SiO_{1.5}(Eu_{0.05}Tb_{0.95})_{0.8}$**.

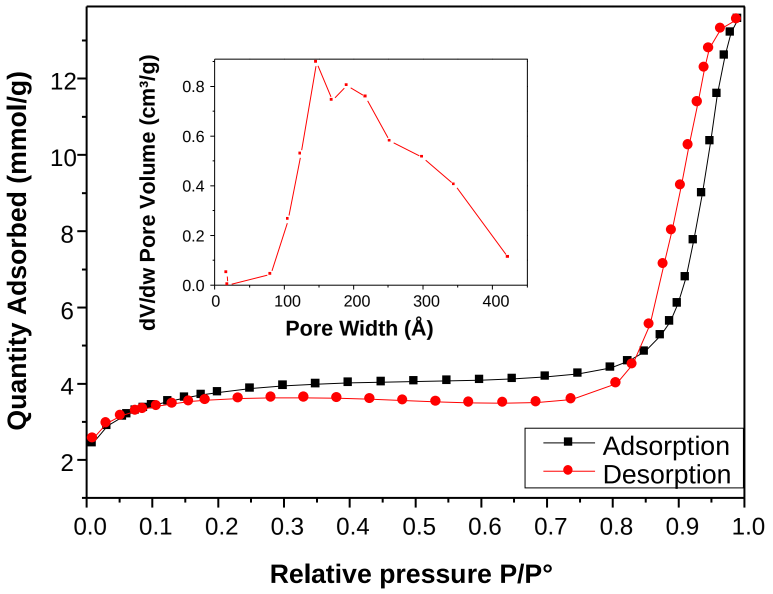

Figure S3. BET adsorption/desorption isotherms for nitrogen adsorption capacity of IONP@SiO_2. Specific surface of 315 $m^2 g^{-1}$ has been estimated by Brunauer−Emmett−Teller (BET) method.

Note: similar shape of adsorption-desorption isotherms has already been obtained for other inorganic core@silica shell nanoparticles prepared by using the micelle inverse method. The observed porosity has been attributed to the interparticle voids.[6],[7]

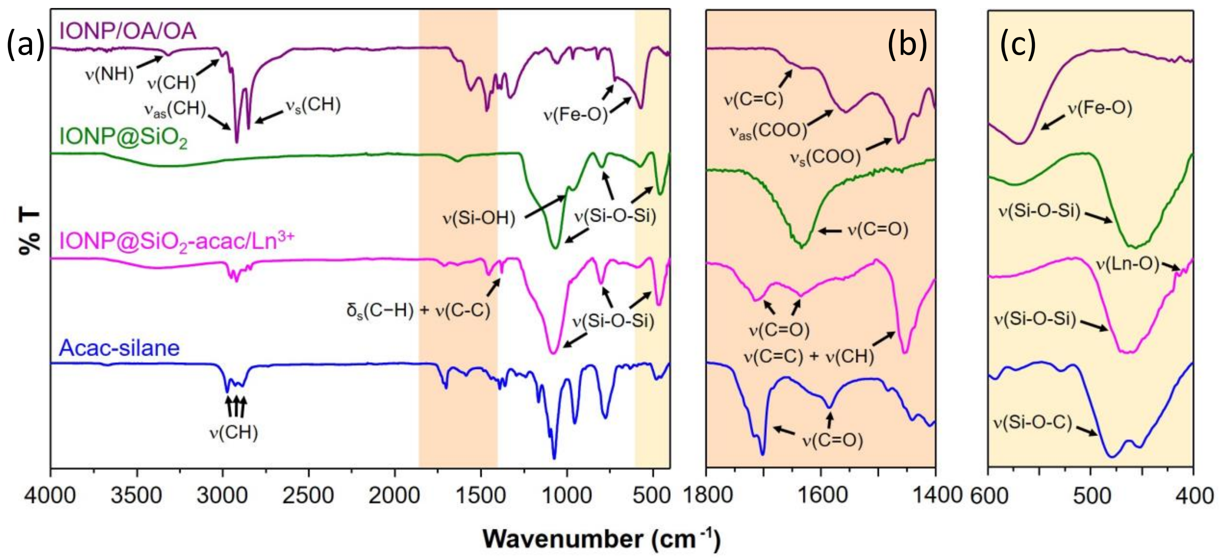

Figure S4. IR spectra of IONP/OA/OA, IONP@SiO_2, IONP@SiO_2-*acac*/Ln^{3+} and *acac*-silane in: (a) the full analyzed range 4000–400 cm^{-1}, (b) the magnification in the window 1800–1400 cm^{-1}, (c) the magnification in the window 600–400 cm^{-1}.

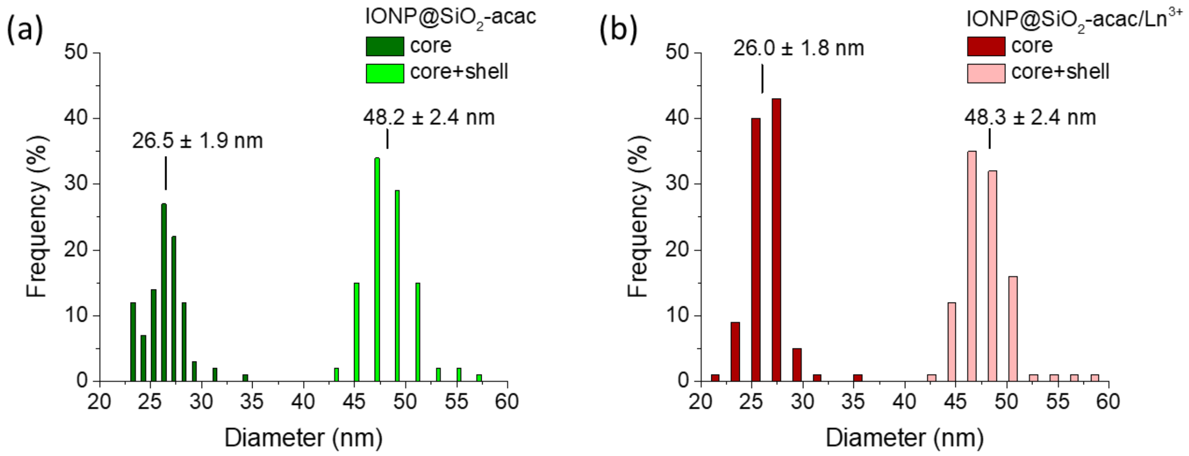

Figure S5. Size distributions of (a) IONP@SiO_2-*acac* and (b) IONP@SiO_2-*acac*/Tb^{3+}/Eu^{3+} (number of counted particles = 100).

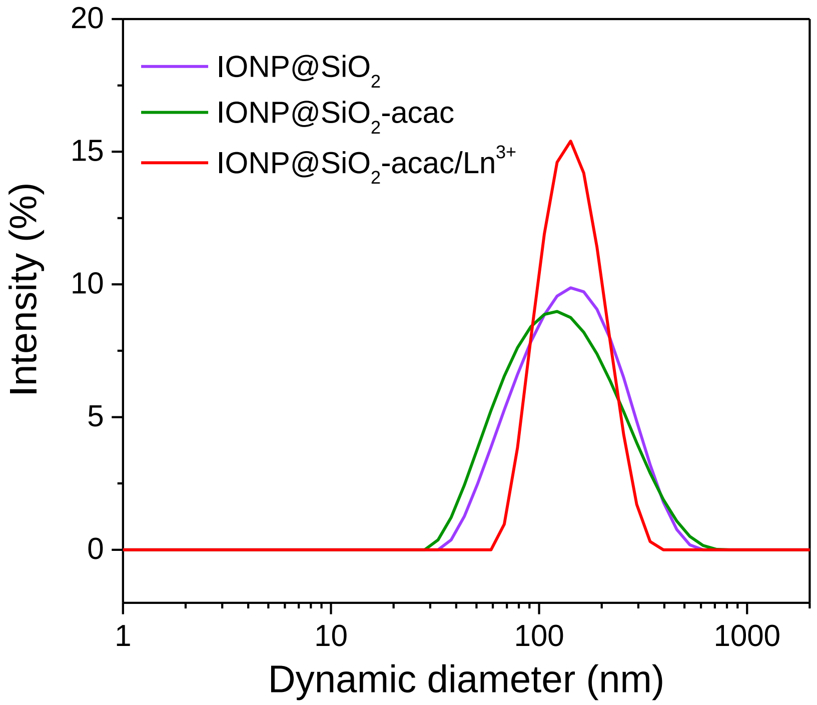

Figure S6. DLS size distribution of IONP@SiO_2 (*ca.*11 nm- silica shell), IONP@SiO_2-*acac* and IONP@SiO_2-*acac*/Ln^{3+} dispersed in ethanol with average dynamic diameters of 159, 150 and 151 nm, respectively.

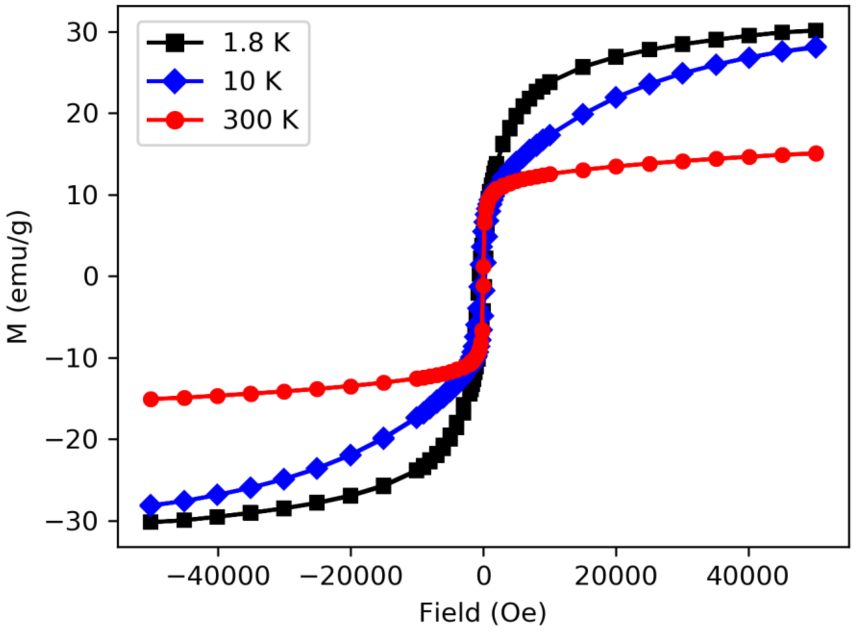

Figure S7. Field dependence of the magnetization for IONP@SiO_2-*acac*/Tb^{3+}/Eu^{3+} nanoparticles at 1.8 K (black square), 10 K (blue diamond) and 300 K (red circle).

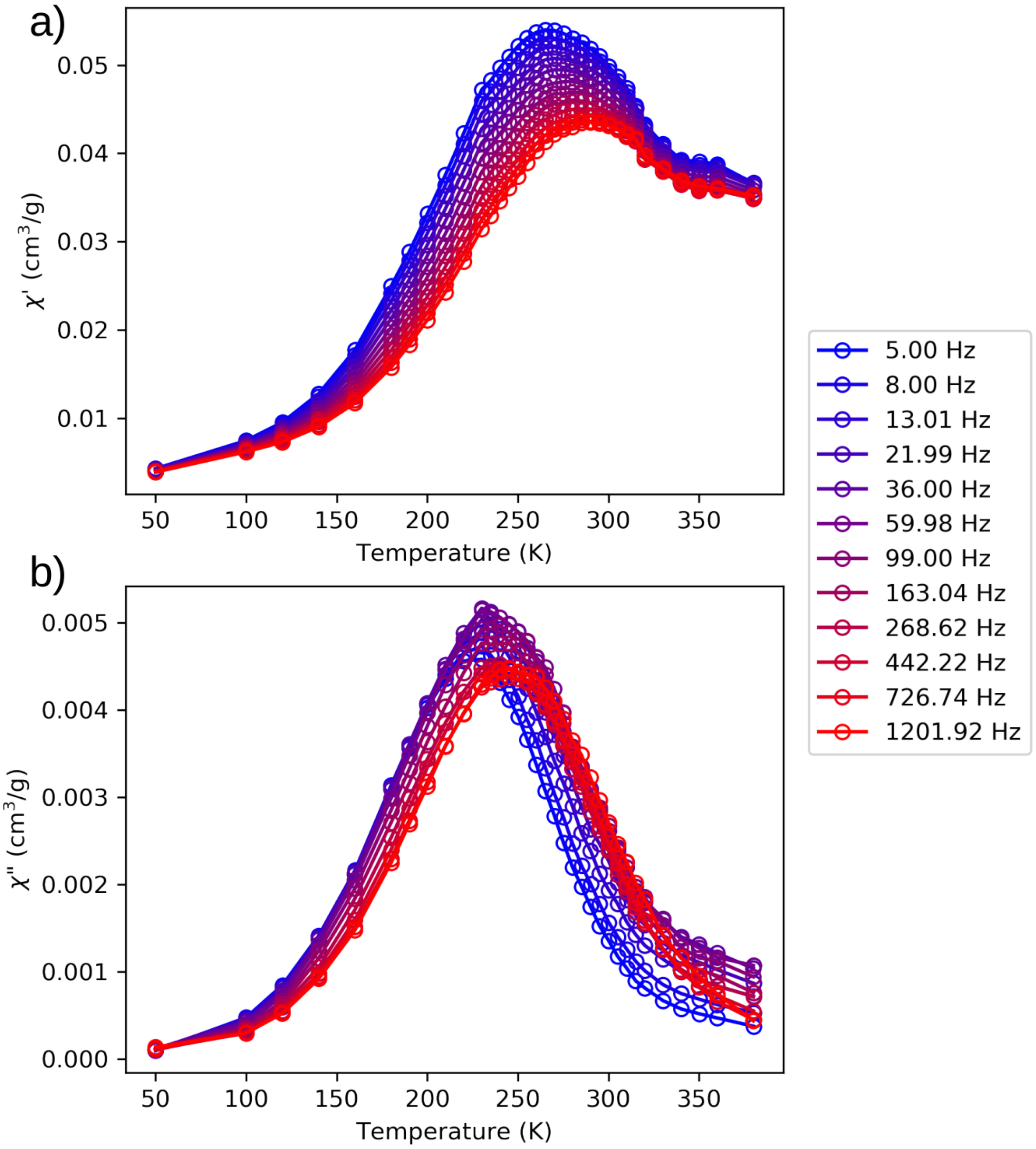

Figure S8. Temperature dependence of: (a) in-phase, χ′, and (b) out-of-phase, χ′′, components of the ac magnetic susceptibility performed at different frequencies for IONP@SiO_2-*acac*/Tb^{3+}/ Eu^{3+} nanoparticles.

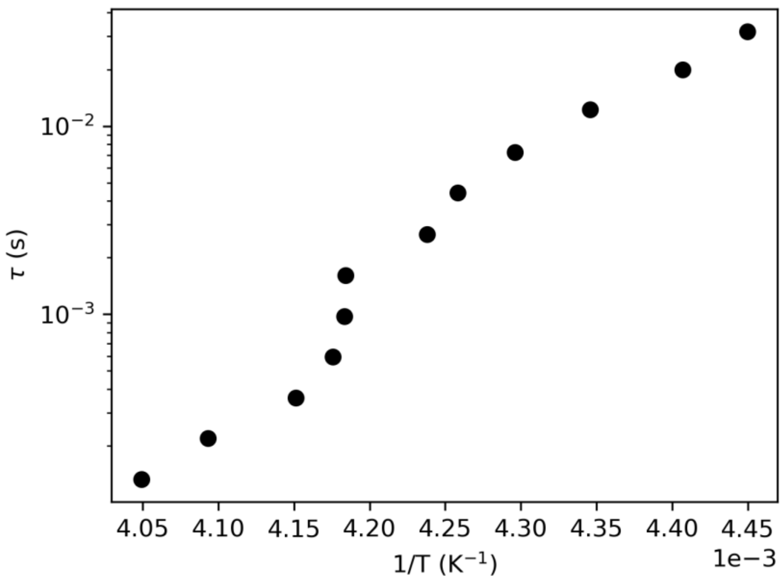

Figure S9. Relaxation time *vs 1/T* curve extracted from the frequency dependence of the out-of-phase component of the magnetic susceptibility.

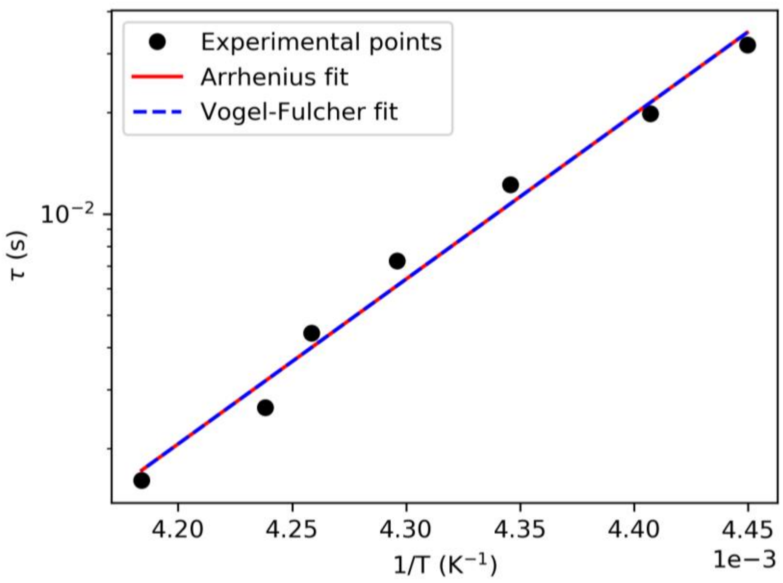

Figure S10. Low temperature region of relaxation time *vs 1/T* curve extracted from the frequency dependence of the out-of-phase component of the ac magnetic susceptibility (black circle) and its theoretical fit by using the Arrhenius model (red line) and the Vogel-Fulcher model (blue dashed line) for IONP@SiO_2-*acac*/Tb^{3+}/Eu^{3+} nanoparticles. *Note*: The fitting of the relaxation time with the Vogel-Fulcher model (equation S2), the values of E_a, τ_0 and T_0 for IONP@SiO_2-acac/Tb^{3+}/Eu^{3+} nanoparticles are equal to 7853 ±345 cm^{-1}, $10^{-23.3\pm0.9}$ s and $5\cdot10^{-11}$ $\pm1\cdot10^{-6}$ K, respectively. The Vogel-Fulcher model, which takes into account the interparticle interactions, leads to conclusion that the dipolar magnetic interactions do not occur for IONP@SiO_2-acac/Tb^{3+}/Eu^{3+} nanoparticles since the values of the energy barrier and the attempt time τ_0 were not

modified in comparison with the Néel law fit. Moreover, the obtained parameter T_0, which represents the impact of the interactions, is very low.

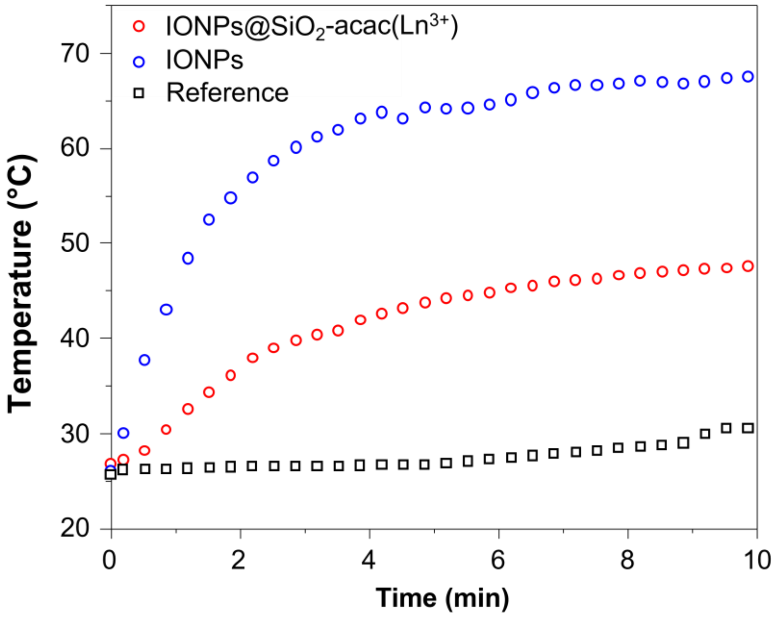

Figure S11. Temperature measurements as a function of time of IONP/OA/OA (red) in cyclohexane and IONP@SiO_2-acac/Ln^{3+} solution in ethanol (blue) and reference (black) subjected to an ac magnetic field (≈ 20 mT at a frequency of 350 kHz.

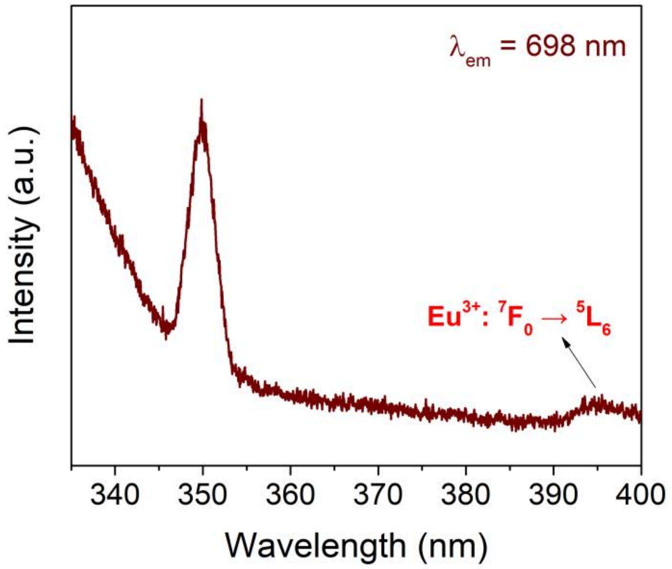

Figure S12. High-resolution spectrum of excitation spectrum monitored at λ_{em} = 698 nm. The peak at 348 nm is the half-order of the monitored wavelength.

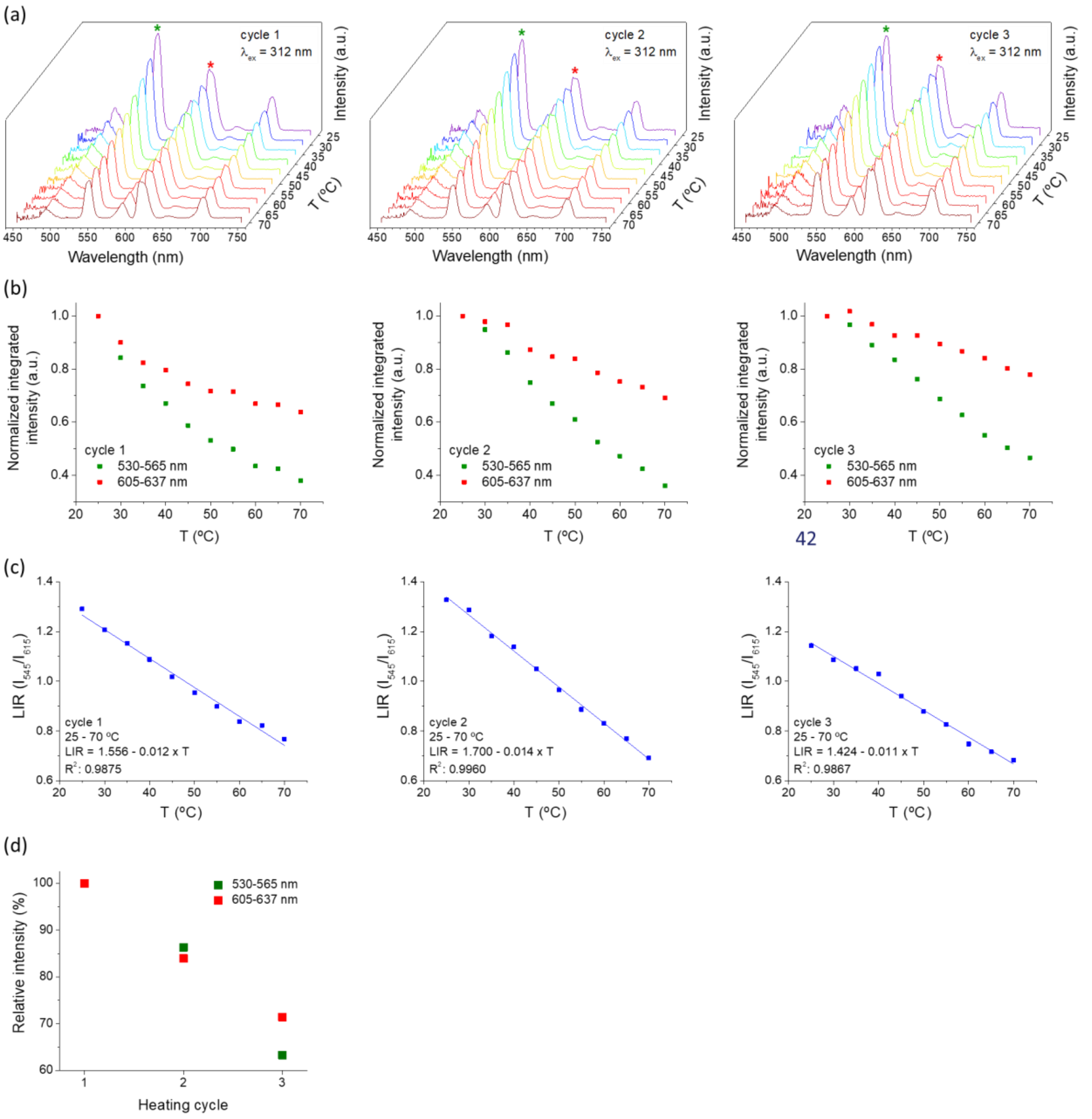

Figure S13. (a) Emission spectra (λ_{ex}=312 nm) at different temperatures for IONP@SiO_2-*acac*/Tb^{3+}/Eu^{3+} obtained upon three consecutive temperature cycles; (b) their respective normalized integrated intensities; and (c) LIR between 545 nm and 615 nm. Wavelength ranges for integrating areas: 530─565 nm (Tb^{3+}: $^5D_4 \rightarrow {}^7F_5$) and 603─637 nm ($Eu^{3+}$: $^5D_0 \rightarrow {}^7F_2$). (d) Relative intensity of green and red emissions at 25 °C along the heating cycles.

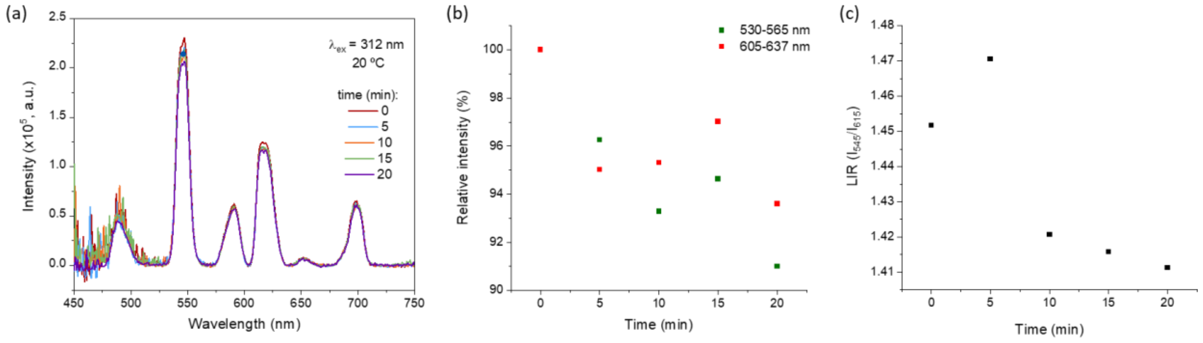

Figure S14. (a) Emission spectra of IONP@SiO_2-*acac*/Ln^{3+} measured after different periods of exposure to excitation light (λ_{ex} = 312 nm). (b) Relative intensities at 545 nm and 615 nm normalized to the spectrum measured at time t = 0. Wavelength ranges for integrating areas: 530—565 nm (Tb^{3+}: $^5D_4 \rightarrow {}^7F_5$) and 603—637 nm ($Eu^{3+}$: $^5D_0 \rightarrow {}^7F_2$). (c) LIR between 545 nm and 615 nm emissions showing the low variation in the thermometric parameter (LIR = 1.43 ± 0.3, relative standard deviation is 1.8 %) along with the time.

References

[1] F. M. Abdel Kerim, H. F. Aly, A. El-Agramy, *Proc. Indian Acad. Sci*. 1977, **85**, No. 6, 559-566.

[2] A. Okamura, S. Nakamura, M. Tanaka, and K. Siratori, *J. Phys. Soc. Jpn.*,1995, **64**, 3484.

[3] A. A. Lykasov, V. V. D'Yachuk, and G. I Sergeev, *Inorg. Mater.*, 1985, **21**, 522.

[4] J. A. Rojas, L. A. Ardila-Rodríguez, M. F. Diniz, M. Gonçalves, B. Ribeiro, and M. C. Rezende, *Materials & Design*, 2019, **166**, 107612.

[5] X. Zhang, L. Clime, H. Roberge, F. Normandin, L. H.Yahia, E. Sacher, and T. Veres, *J. Phys. Chem. C*, 2011, **115**, 1436.

[6] H. Peng, T. Dong, L. Zhang, C. Wang, W. Liu, J. Bao, X. Wang, N. Zhang, Z. Wang, P. Wu, P. Zhang, S. Dai, *Appl. Catal. B*, 2019, **256**, 117807

[7] J. Mielby, A. J. Kunov-Kruse, S. Kegnæs, *J. Catal.* 2017, **345**, 149–156.
